# Functional isolation, culture and cryopreservation of adult human primary cardiomyocytes

**DOI:** 10.1038/s41392-022-01044-5

**Published:** 2022-07-27

**Authors:** Bingying Zhou, Xun Shi, Xiaoli Tang, Quanyi Zhao, Le Wang, Fang Yao, Yongfeng Hou, Xianqiang Wang, Wei Feng, Liqing Wang, Xiaogang Sun, Li Wang, Shengshou Hu

**Affiliations:** 1 State Key Laboratory of Cardiovascular Disease, Fuwai Hospital, National Center for Cardiovascular Diseases, Chinese Academy of Medical Sciences and Peking Union Medical College, Beijing, China; 2 Shenzhen Key Laboratory of Cardiovascular Disease, Fuwai Hospital Chinese Academy of Medical Sciences, Shenzhen, Shenzhen, China; 3 Department of Cardiac Surgery, Fuwai Hospital, National Center for Cardiovascular Diseases, Chinese Academy of Medical Sciences and Peking Union Medical College, Beijing, China; 4 Present Address: 18 Jinma Industrial Park, Fangshan District, Beijing, China

**Keywords:** Cardiology, Cell biology

## Abstract

Cardiovascular diseases are the most common cause of death globally. Accurately modeling cardiac homeostasis, dysfunction, and drug response lies at the heart of cardiac research. Adult human primary cardiomyocytes (hPCMs) are a promising cellular model, but unstable isolation efficiency and quality, rapid cell death in culture, and unknown response to cryopreservation prevent them from becoming a reliable and flexible in vitro cardiac model. Combing the use of a reversible inhibitor of myosin II ATPase, (-)-blebbistatin (Bleb), and multiple optimization steps of the isolation procedure, we achieved a 2.74-fold increase in cell viability over traditional methods, accompanied by better cellular morphology, minimally perturbed gene expression, intact electrophysiology, and normal neurohormonal signaling. Further optimization of culture conditions established a method that was capable of maintaining optimal cell viability, morphology, and mitochondrial respiration for at least 7 days. Most importantly, we successfully cryopreserved hPCMs, which were structurally, molecularly, and functionally intact after undergoing the freeze-thaw cycle. hPCMs demonstrated greater sensitivity towards a set of cardiotoxic drugs, compared to human-induced pluripotent stem cell-derived cardiomyocytes (hiPSC-CMs). Further dissection of cardiomyocyte drug response at both the population and single-cell transcriptomic level revealed that hPCM responses were more pronouncedly enriched in cardiac function, whereas hiPSC-CMs responses reflected cardiac development. Together, we established a full set of methodologies for the efficient isolation and prolonged maintenance of functional primary adult human cardiomyocytes in vitro, unlocking their potential as a cellular model for cardiovascular research, drug discovery, and safety pharmacology.

## Introduction

Cardiovascular diseases continue to be the leading cause of mortality worldwide, claiming more than 17 million lives annually, and contribute to an enormous economic burden.^1^ Therefore, both research into disease mechanisms and cardiovascular drug discovery are fundamental to alleviating this increasing burden.

Despite major advances in cardiac modeling in the past decades, cardiovascular research and drug discovery still suffer from a lack of adequate cardiac models.^2^ At present, rodent models remain the desired method for in vivo studies, whereas human pluripotent stem cell (hPSC)-derived cardiomyocytes (hPSC-CMs) have become one of the most popular in vitro models in recent years.^3–7^ hPSC-CMs are now widely used in both basic research^8–11^ and preclinical safety pharmacology,^12–15^ and immensely improved our understanding of the physiology, pathology, and pharmacology of the human heart. However, hPSC-derived cells are known to exhibit immature phenotypes in many aspects, including cell structure, gene expression, metabolism, and electrophysiology, at which current efforts are directed.^16,17^ By contrast, human primary cardiomyocytes (hPCMs), which are directly isolated from human heart tissue, possess literally all the desired elements of a cellular model (e.g., native structure and function, patient-specific genetic and epigenetic information, etc.), and thus is in theory an ideal cellular model.^18^ Emerging studies are using hPCMs as models to predict drug response, particularly arrhythmia and contraction deficiencies.^18,19^ A set of 26 inotropes (17 positive and 9 negative) was tested and their mechanisms of action were explored, in adult hPCMs by measuring and analyzing a panel of 12 contractility parameters.^20^ Importantly, hPCMs demonstrated lower rates of false positive and false negatives than hiPSC-CMs in screening for multi-ion channel blocking drugs.^21^ As such, the US Food and Drug Administration is moving towards including hPCMs as a human-relevant platform in the drug discovery pipeline.^22^ However, current hPCM-based studies are restricted to the use of acutely isolated cells, and are limited in cell number, because reliable and reproducible methods for hPCM isolation and culture is lacking.

Technical difficulties in the isolation, culture and scaled production of hPCMs have hampered its use in cardiovascular research and drug discovery. The isolation of hPCMs from human myocardial specimens, which lack intact vasculature for perfusion, has relied on the mechanical separation of tissue chunks followed by enzymatic dissociation of cells. This method, termed ‘chunk digestion’, often results in unpredictable isolation quality, owing to the fragility of hPCMs and uneven enzyme penetration into the tissue. Although considerable attempts have been made to optimize isolation techniques, existing methods demonstrated low-yield and unstable quality (cell survival rate of 1–55%, depending on the literature),^23–28^ or isolated hPCMs failed to exhibit proper electrophysiology, and were not amenable to long-term in vitro culture, let alone cryopreservation.^29^ Therefore, few studies have utilized hPCMs beyond acute isolation. In addition, to the best of our knowledge, there has been no attempt at cryopreserving adult primary cardiomyocytes, whether from animals or humans. Yet filling these technical gaps is pivotal to establishing a reliable hPCM platform for both basic and translational research.

In this study, we developed a methodology for the efficient isolation, primary culture, and cryopreservation of adult human primary cardiomyocytes (hPCMs). Systematic evaluation of functional and molecular features of these hPCMs demonstrated the reliability and robustness of established methods. In addition, to demonstrate the potential application of the hPCM cell model, we show that hPCMs could be exploited to predict the cardiotoxicity of drugs, evidenced by their higher sensitivity compared to human-induced pluripotent stem cell-derived cardiomyocytes. Single-cell and bulk drug response at the transcriptome level unveiled inherent distinctions between primary cardiomyocytes and their in vitro-derived counterparts. Overall, hPCMs responded in a cardiac function-dependent manner, while hiPSC-CMs were more inclined to respond with genes related to cardiac development. Together, this set of methods, particularly in vitro culture and cryopreservation of hPCMs, are projected to enable genetic manipulation, reliable pharmacological assessments, and high-throughput screening, expanding the toolkit of cardiovascular research and the pharmaceutical industry.

## Results

### Blebbistatin improves hPCM isolation efficiency

**Fig. 1:**
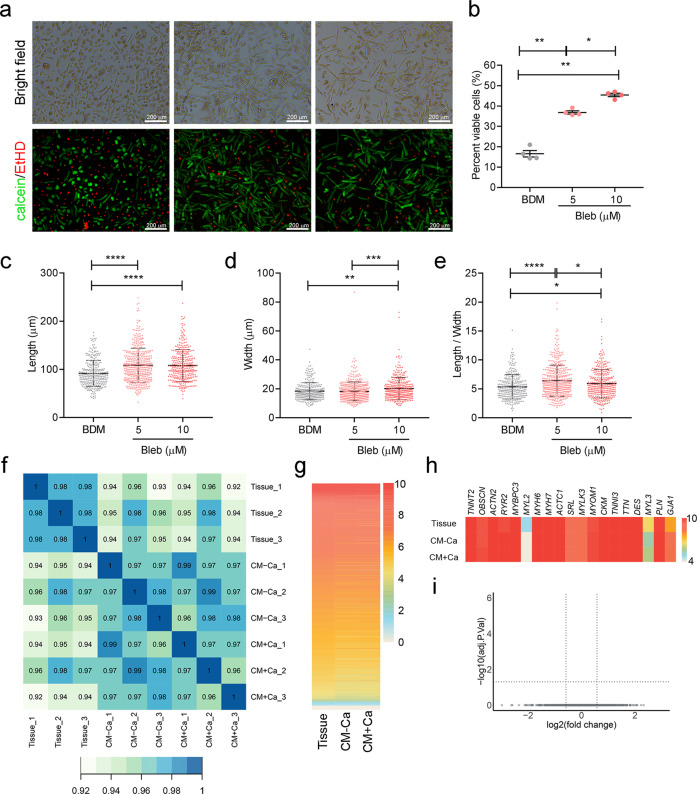
Blebbistatin improves the isolation efficiency of adult human primary cardiomyocytes (hPCMs) without changing gene expression. **a** Freshly isolated hPCMs were stained for cell viability using calcein AM (green, live) and ethidium homodimer-1 (red, dead). Scale bar = 200 μm.Results are representative of four independent experiments. **b** Quantification of cell viability in **a**. Data were mean ± SEM, one-way repeated measures ANOVA. *P < 0.05, **P < 0.005. **c**–**e** Quantification of **ce**ll lengths (**c**), cell widths (**d**), and length-to-width ratios (**e**) in **a**. Data were mean ± SD, one-way ANOVA. *P < 0.05, **P < 0.005, ***P < 0.0005, ****P < 0.0001. **f** Spearman’s correlation between paired tissue and cell samples (*n* = 3 biological repeats, denoted by the number at the end of sample name). Tissue, myocardial tissue specimen; CM-Ca, isolated hPCMs prior to calcium reintroduction; CM + Ca, isolated hPCMs after calcium reintroduction. **g** Ranking of mean expression of genes. **h** Expression of CM marker genes. **i** Volcano plot of differentially expressed genes (DEGs) between hPCMs before and after calcium reintroduction

The isolation of cardiomyocytes from human myocardial specimens is usually achieved through the enzymatic digestion of tissue chunks. Myosin ATPase inhibitor 2,3-butanedione monoxime (BDM) is normally added to the digestion buffer to minimize cardiomyocyte energy expenditure and was shown to improve cell viability.^30^ However, the viability and quality of hPCMs isolated using existing protocols still fall short of establishing hPCMs as a reliable cell model. (-)-Blebbistatin (Bleb), another myosin ATPase inhibitor, has been previously reported in mice to extend the culture life of cardiomyocytes,^31,32^ but is not routinely used in the context of cardiomyocyte isolation. Therefore, we tested the effect of Bleb on the isolation of hPCMs from left atrial appendages. Compared to BDM (20 mM) control, the application of Bleb at 5 μM resulted in a 2.2-fold increase in cell viability, while a concentration of 10 μM achieved a 2.74-fold increase (*P* < 0.01) (Fig. 1a, b). Cardiomyocyte deterioration is marked by cell shape distortions, such as cell shrinkage, following isolation, resulting in compromised cardiomyocyte cell size. Bleb used at 5 μM increased cell length by 18.5% compared to BDM control, whereas further increasing the concentration to 10 μM did not show any further advantage in maintaining cell length (Fig. 1c). However, 5 μM Bleb failed to confer any advantage over BDM with respect to retaining cell width, whereas 10 μM Bleb exhibited a drastic improvement, increasing average cell widths by ~10% (Fig. 1d). Therefore, while both 5 and 10 μM exhibited significant improvements in the length-to-width ratios of hPCMs, the latter was less prominent (Fig. 1e). Together, these data indicated that Bleb was more effective than BDM in cardiomyocyte isolation, by increasing cell viability and maintaining cell morphology.

Many chunk digestion protocols suggest continuous gassing of oxygen into the digestion solution throughout isolation to ensure sufficient oxygenation and to reduce cell death.^25,33,34^ However, according to our observations, oxygenation did not appear advantageous, and may even be damaging to cells. Compared to non-oxygenated control, cardiomyocytes digested under constant gassing displayed lower viability, as well as paradoxically lower ATP contents (Supplementary Fig. S1a, b). Therefore, based on these findings, we avoided continuous oxygenation during the isolation procedure. To determine the effectiveness of our protocol, we calculated cell yields from 376 surgeries performed at Fuwai Hospital during the years 2020–2021. We achieved an average hPCM yield of 3.28 × 10^6^ cells/gram of tissue (Supplementary Fig. 1c), highlighting the robustness of this approach.

Ventricular hPCMs are regarded as more pertinent to many cardiac diseases compared to hPCMs from left atrial appendages. To evaluate the versatility of our methodology, we applied it to ventricular tissue of different health states, including healthy, hypertrophic obstructive cardiomyopathy, and end-stage heart failure. Isolation of these tissues produced satisfactory cell morphology and viability (Supplementary Fig. 1d).

### Gene expression of cardiomyocytes is minimally affected during isolation

The greatest concern surrounding primary cell isolation is dissociation-induced artifacts in gene expression. To this end, we used RNA-seq to evaluate how well-isolated hPCMs represented cells in vivo, by comparing isolated cells with matched myocardial tissue. To subtract the influence of non-CM cell types in myocardial tissues on gene expression analysis, we applied CIBERSORTx to impute cell type-specific gene expression, yielding CM-specific gene expression profiles (Supplementary Fig. S2a). Following logarithmic conversion, total CM gene expression was comparable among samples (Supplementary Fig. S2b). As expected, tissue-CM samples were more similar to one another than to isolated CMs (Fig. 1f). However, the correlation between tissue-CMs and corresponding isolated CM samples was also satisfactory (correlation coefficient ≥0.94). Globally, gene expression ranked by their expression level was not significantly different between tissue-CMs and isolated CMs (Fig. 1g). Closer inspection of CM-specific marker genes demonstrated comparable gene abundances, except for *MYL2*, *MYL3*, and *GJA1*, whose changes fold changes were nonetheless insignificant (|Log_2_FC| < 0.58) (Fig. 1h). These findings suggested that the isolation procedure minimally perturbed the cardiomyocyte transcriptome, rendering isolated hPCMs suitable for gene expression analyses.

Restoring calcium to physiological concentrations is the final step in cardiomyocyte isolation, which may induce hypercontraction of cells, thereby compromising cell viability.^35^ To this end, we examined the viability of hPCMs before and after calcium reintroduction. Although data showed a slight decrease, it did not reach statistical significance (*P* = 0.1250, Supplementary Fig. S2c). Despite unchanged cell viability, we further evaluated whether this step induced any changes in gene expression. We exploited our RNA-seq data to focus on cells prior to and after calcium reintroduction. Analyses of gene expression failed to identify any gene with significant differential expression between cardiomyocytes depleted of calcium and those that were not (Fig. 1i). This finding indicated that calcium reintroduction did not disrupt global gene expression in Bleb-isolated cells and that calcium reintroduction may be dispensable for hPCM gene expression analysis.

### Functional characterization of freshly isolated hPCMs

**Fig. 2:**
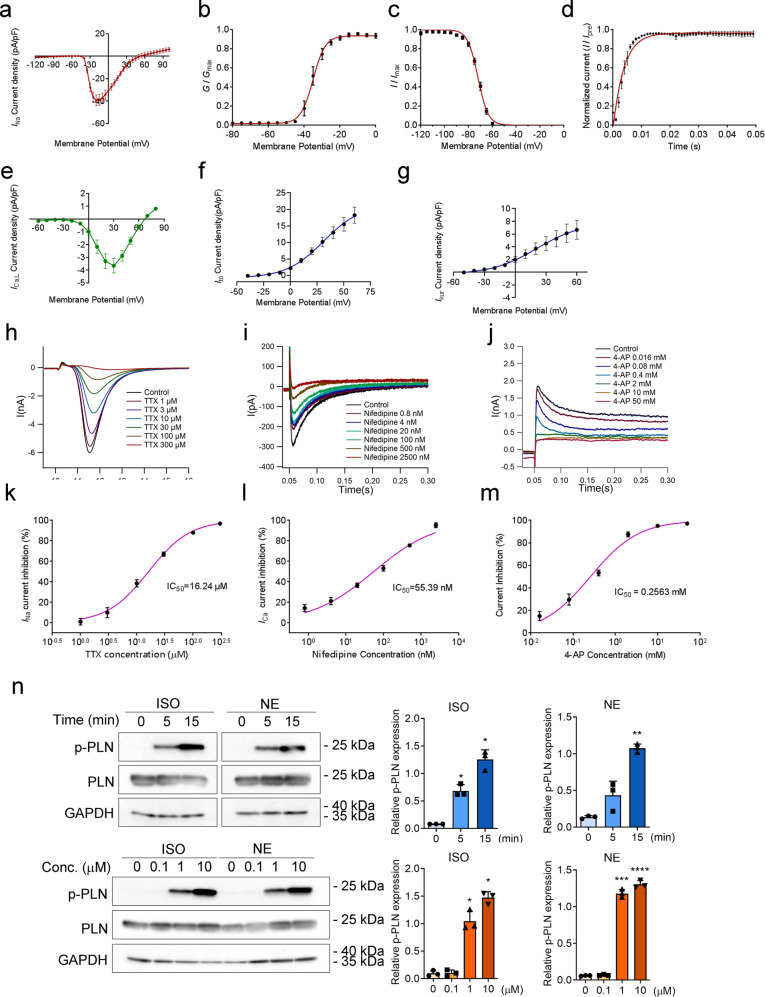
Functional integrity of freshly isolated hPCMs. **a**–**d** Voltage-dependent kinetics of sodium currents. Current–voltage (I–V) relation curve (**a**), Voltage-dependent activation (**b**), inactivation (**c**), and recovery (**d**) curves of *I*_Na_ (*n* = 10). **e**–**g** I–V relationships for *I*_Ca, L_ (*n* = 4) (**e**), *I*_to_ (*n* = 7) (**f**), and *I*_kur_ (*n* = 8) (**g**). **h**–**m** Dose-dependent current inhibition of *I*_Na_ (**h**, **k**), *I*_Ca, L_ (**i**, **l**), and *I*_to_ (**j**, **m**). Data were mean ± SEM. **n** Phosphorylation of phospholamban (PLN) in response to isoproterenol (ISO) and norepinephrine (NE) in a time- and dose-dependent manner (upper and lower panels, respectively) in hPCMs. GAPDH was used as a loading control. For time dependency experiments, stimulants were used at a concentration of 10 μM, while for the dose-dependency experiment, agonists were applied for 45 min. The blots shown are representative of three independent experiments. Quantifications of blots are shown on the right. Data were mean ± SD, one-way repeated measures ANOVA. *P < 0.05, **P < 0.005, ***P < 0.0005, ****P < 0.0001

Next, we tested whether isolated cardiomyocytes were functionally competent. First, we used patch clamping to characterize major membrane currents and their responses to specific inhibitors. Sodium currents were elicited by a series of depolarizing test potentials between −120 to +100 mV with 30 mV steps at a holding potential of −120 mV. The peak density of sodium current was at −20 mV, and the mean maximal peak *I*_Na_ normalized to cell capacitance was −39.27 ± 14.50 pA/pF (*n* = 10) (Fig. 2a). We also characterized the voltage-dependent steady-state activation and inactivation curves of sodium currents. The voltage at half activation (*V*_1/2_) was −35.07 ± 0.87 mV (*n* = 10), with a mean slope factor (*k*) of 3.43 ± 0.76, whereas the *V*_1/2_ for inactivation −72.13 ± 0.54 mV (*n* = 10), and the slope factor was 4.30 ± 0.47 (Fig. 2b, c). Time-dependent recovery of sodium currents from inactivation was assessed using a paired-pulse protocol. The mean recovery time constant of sodium currents was 3.83 ± 0.35 ms (*n* = 10) (Fig. 2d). Likewise, L-type Ca^2+^ currents (*I*_Ca,L_) currents were elicited at potentials between −60 to +90 mV, with 30 mV steps at a holding potential of −80 mV. The I–V relationship showed that the peak density of calcium currents occurred at 30 mV, and the mean maximal peak *I*_Ca,L_ normalized to cell capacitance was −3.66 ± 0.98 pA/pF (*n* = 4) (Fig. 2e). The *V*_1/2_ for inactivation of *I*_Ca,L_ was −13.04 ± 0.93 mV, with a mean slope factor of 5.96 ± 0.76 (*n* = 4) (Supplementary Fig. S3a). The mean recovery time constant of *I*_Ca,L_ was 135.2 ± 6.6 ms (*n* = 4) (Supplementary Fig. S3b). We further characterized the current–voltage (I–V) relationships of two major potassium currents in freshly isolated hPCMs. For the 4-aminopyridine (4-AP)-sensitive, non-Ca^2+^-dependent transient outward potassium current (*I*_to_), its current density averaged −0.13 ± 0.24 pA/pF at −40 mV, 2.17 ± 1.06 pA/pF at 0 mV, and 12.96 ± 4.32 pA/pF at +40 mV (Fig. 2f). The ultrarapid outward current *I*_kur_ is the other major repolarizing current in the human atrium.^36^ *I*_kur_ density averaged 0.24 ± 0.16 pA/pF at −40 mV, 2.00 ± 1.23 pA/pF at 0 mV, and 5.28 ± 3.39 pA/pF at +40 mV (Fig. 2g). Together, these data demonstrate proper ionic currents of isolated hPCMs.

To further validate the functionality of cardiomyocytes, we tested whether these currents responded correctly to specific channel inhibitors. To this end, we used tetrodotoxin (TTX), nifedipine, and 4-aminopyridine (4-AP) to respectively inhibit Na^+^ (*I*_Na_), Ca^2+^ (*I*_Ca_), and K^+^ (*I*_to_) currents (Fig. 2h–j). The half-maximal inhibitory concentration (IC_50_) values for TTX, nifedipine, and 4-AP were 16.24 μM, 55.39 nM, and 0.2563 mM, respectively, which were similar to reports in the literature^37–39^ (Fig. 2k–m). Taken together, isolated hPCMs exhibited normal electrophysiology, indicating their potential use as models to evaluate drugs that affect the electrical properties of cardiomyocytes.

We next used isoproterenol and norepinephrine to examine whether cells responded to neurohormonal stimulation. Both stimuli caused time- and dose-dependent increases in the phosphorylation of phospholamban, an important regulator of calcium handling in cardiomyocytes (Fig. 2n), demonstrating that cells retained functional adrenergic signaling following isolation.

To determine whether the isolation procedure disrupted mitochondrial health, we assessed mitochondrial membrane potential by JC-1 staining. Freshly isolated hPCMs exhibited bright red fluorescence, indicating that cells had healthy mitochondria (Supplementary Fig. S3c). Taken together, acutely isolated cells exhibited proper gene expression and cardiomyocyte function.

### Optimization of hPCM cell culture conditions

**Fig. 3:**
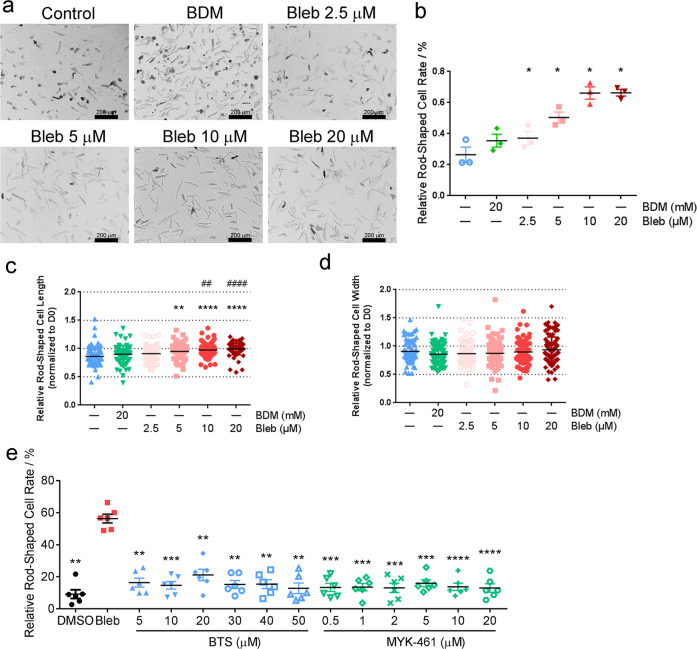
Blebbistatin improves hPCM culture. **a** Live-cell imaging (D5) of hPCMs cultured with different concentrations of Bleb. Images are representative of three independent experiments. **b** Quantification of the relative percentage of rod-shaped cells in **a**. **c**, **d** Tracking of cell length (**c**) and cell width (**d**) changes by D5, normalized to D0. **e** Comparison of the effect of Bleb with two other myosin inhibitors, BTS and MYK-461 (D7). Data were mean ± SEM (**b**, **e**) or mean ± SD (**c**, **d**), statistical significance was calculated by one-way repeated measures ANOVA (**b**, **e**) or ordinary one-way ANOVA (**c**, **d**). For **b**–**d**, *P < 0.05, **P < 0.01, ***P < 0.0005, ****P < 0.0001, compared to control; ^##^*P* < 0.01, ^####^*P* < 0.0001, compared to BDM^.^ For **e** **P < 0.01, ***P < 0.0005, ****P < 0.0001, compared to Bleb

At present, there is a lack of efficient culture methods to maintain the differentiated state of isolated adult mammalian cardiomyocytes for a prolonged period of time.^24^ The effect of Bleb during isolation inspired us to test its use in extended cardiomyocyte culture. As anticipated, the addition of Bleb dose-dependently increased cellular survival compared to control and BDM at both D5 and D7 (Fig. 3a, b and Supplementary Fig. S4a, b). Importantly, by tracking individual cells with live-cell imaging, we observed that Bleb was capable of maintaining the elongated morphology of cardiomyocytes (manifested as cell length changes in culture), which also demonstrated dose-dependency (Fig. 3c, d and Supplementary Fig. S4c, d). For the maintenance of viability and morphology, 10 and 20 μM both exhibited significant advantages. However, given the reported poor solubility of Bleb and other concomitant problems, such as cellular toxicity, at concentrations above 10 μM,^40^ we decided to use Bleb at 10 μM for all further experiments.

To determine whether the pro-survival effect was specific to Bleb, or general to myosin ATPase inhibitors, we tested two potent inhibitors, *N*-benzyl-*p*-toluenesulfonamide (BTS) and mavacamten (MYK-461), the latter of which has recently completed its phase 3 clinical trial for the treatment of hypertrophic cardiomyopathy. Following 1 week in culture, neither BTS nor MYK-461 exhibited any significant improvement in cellular survival compared to DMSO control, whereas Bleb clearly outperformed all conditions (Fig. 3e). We further applied para-amino-blebbistatin (PAB), a highly soluble, non-phototoxic and non-fluorescent blebbistatin derivative^40^ in the culture of hPCMs. Mid- to high-concentration PAB produced a survival effect similar to that of Bleb (*P* > 0.05), suggesting that it may be used as an alternative to Bleb (Supplementary Fig. S4e).

We then optimized additional culture conditions, such as basal medium and cell attachment surface. M199 is most commonly used as the medium for cardiomyocyte culture, followed by DMEM. To determine the most suitable basal medium, we compared the effects of different media on cellular survival in culture. Following 1 week in culture, none of the tested media demonstrated significantly superior survival advantage (Supplementary Fig. S4f). Nonetheless, due to the modestly greater survival rate of MEM at each time point, we decided on the use of MEM as the basal medium for cardiomyocyte culture. We also found that cardiomyocytes benefited modestly from the further addition of GlutaMAX and HEPES (Supplementary Fig. S4g). Finally, we determined the optimal surface coating material and concentration for proper cardiomyocyte attachment. Consistent with the literature, we identified laminin as the most suitable substrate for cardiomyocyte growth, followed by Matrigel (Supplementary Fig. S4h). However, an unusually high concentration of laminin (i.e., 200 μg/ml) was required to facilitate attachment and ensure adequate cell survival. As a proof of concept, we applied these culture conditions to ventricular cardiomyocytes isolated from healthy myocardium and from the left ventricular free wall of a heart transplant recipient (Supplementary Fig. S5). Both normal and diseased ventricular hPCMs retained the characteristic rod-shaped morphology in culture (Supplementary Fig. S5).

### Structural and functional assessment of human cardiomyocytes in culture

**Fig. 4:**
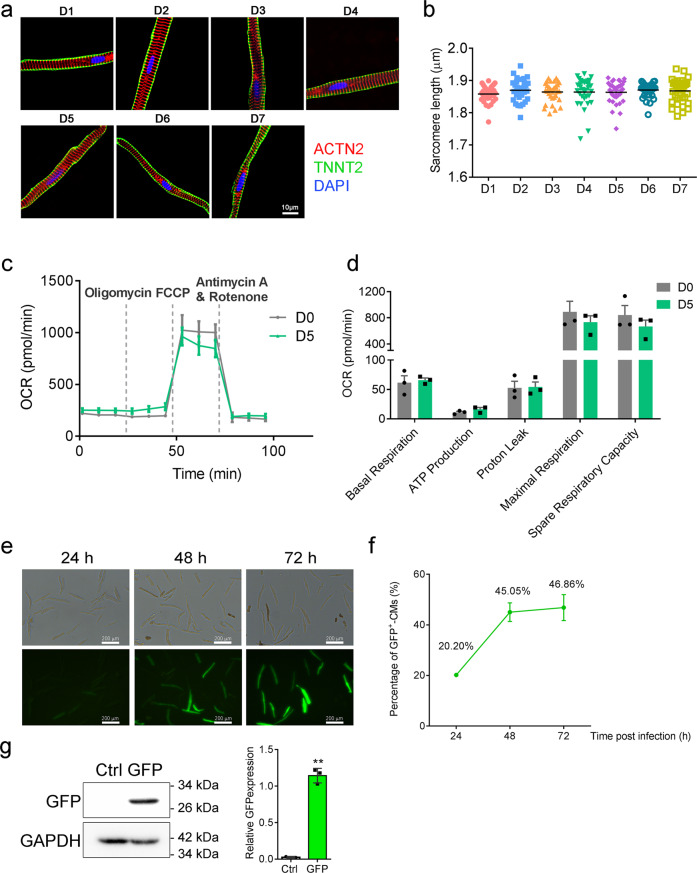
Structural and functional evaluation of hPCMs in culture. **a** Immunofluorescence of cardiac markers ACTN2 (red) and TNNT2 (green) during hPCM culture until D7. Images are representative of three independent experiments. **b** Quantification of sarcomere lengths in **a**. **c** Measurement of oxygen consumption rates of freshly isolated hPCMs (D0) and cultured hPCMs (D5) by Seahorse assay. Data were mean ± SEM, from three independent experiments (**d**) Quantification of **c**. **e** Infection of cultured hPCMs with GFP-expressing adenovirus. Images are representative of three independent experiments. **f** Quantification of GFP-expressing cells for the first 72 h post-infection. Data were mean ± SEM. **g** GFP protein expression analysis by western blotting. hPCMs were transduced by either GFP-expressing adenovirus (GFP) or empty vector control (Ctrl), and protein expression was analyzed by western blotting (left). GAPDH was used as an internal control. Results are representative of three independent experiments. Right: quantification of western blots. **P < 0.01, paired *t*-test

Next, we assessed the structural integrity of Bleb-cultured cardiomyocytes by immunofluorescence. While mild myofilament atrophy was evident, cells that survived culture still displayed regular sarcomere arrangement, and sarcomere lengths did not alter significantly over the first week in culture (Fig. 4a, b).

Metabolic integrity is key to cardiomyocyte function. To evaluate whether hPCMs in culture maintained proper metabolism, we measured oxygen consumption rates using a Seahorse Analyzer. After 5 days in culture, hPCMs exhibited no significant differences in basal respiration, ATP production, proton leak, maximal respiration, and spare respiratory capacity, compared to freshly isolated hPCMs (D0) (Fig. 4c, d). Of note, both acutely isolated and cultured hPCMs exhibited an oligomycin-insensitive phenotype, which has been previously observed.^41,42^

We further tested whether cultured hPCMs were amenable to genetic manipulations. As a proof of concept, we infected hPCMs with adenovirus expressing GFP and observed green fluorescence for the first 72 h. The number of GFP-positive hPCMs rose from 20.20% at 24 h to 45.05% at 48 h and remained relatively stable until 72 h (46.86%) (Fig. 4e, f). Western blotting confirmed adenovirus-mediated expression of GFP in hPCMs (Fig. 4g).

Together, these data showed that hPCMs in culture were structurally and metabolically intact, and were compatible with adenovirus-mediated gene expression.

### Cryopreservation of human cardiomyocytes

**Fig. 5:**
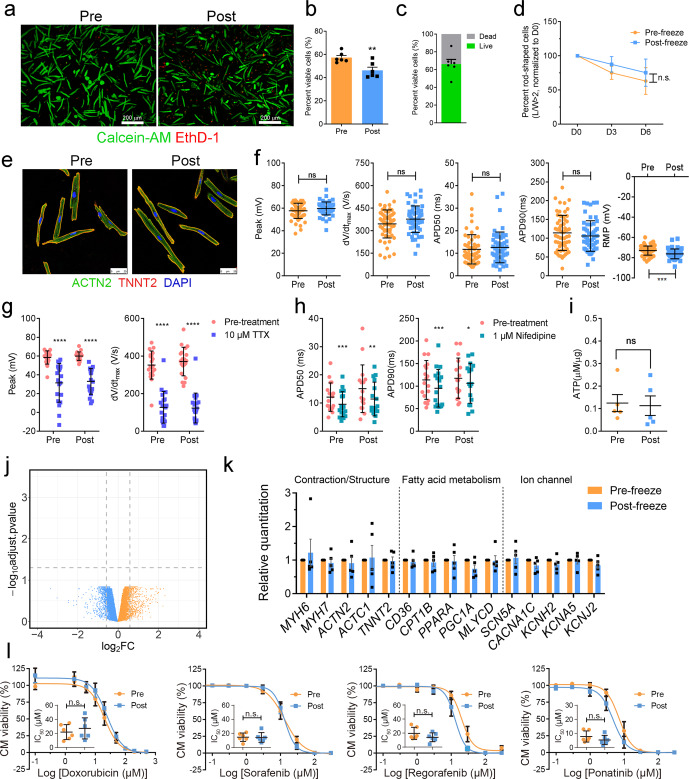
Cryopreservation of hPCMs. **a** Cell viability staining of fresh and cryopreserved hPCMs. Images are representative of six independent experiments. Scale bar = 200 μm. **b** Quantification of **a**, **P < 0.005, paired two-tailed Student’s *t*-test. **c** Cell recovery rate after cryopreservation, quantified from **a**. **d** Live-cell imaging of hPCMs before and after cryopreservation. Cells were cultured for 6 days, and the number of rod-shaped cells was quantified as a percentage of the starting number on D0. Data were representative of four independent experiments. n.s. not significant (*P* > 0.05) paired two-tailed Student’s *t*-test. **e** Representative confocal micrographs of hPCMs before and after cryopreservation. Red, ACTN2; green, TNNT2; blue, DAPI. Data were representative of seven independent experiments. Scale bar = 25 μm. **f** Baseline action potential recordings of hPCMs before and after cryopreservation. Whole-cell patch clamping was performed on hPCMs in culture. *n* = 5 patients, n.s. *n*ot significant (*P* > 0.05), ***P < 0.001, unpaired two-tailed Student’s *t*-test. **g**, **h** Action potential recordings of fresh and cryopreserved hPCMs in response to 10 μM TTX (**g**) or 1 μM nifedipine (**h**). *n* = 5 patients, ****P < 0.0001, ***P < 0.001, **P < 0.01, *P < 0.05, paired two-tailed Student’s *t*-test. **i** ATP measurement of pre- and post-freeze hPCMs. ATP was measured using a luminescent ATP detection kit and normalized to total cellular protein. Data were representative of five independent experiments. n.s. not significant (*P* > 0.05) paired two-tailed Student’s *t*-test. **j** Volcano plot showing DEGs between hPCMs before and after cryopreservation, as analyzed by RNA sequencing (RNA-seq). Data were representative of four independent experiments. **k** Real-time quantitative PCR (qPCR) validation of gene expression of hPCMs before and after cryopreservation. Data were representative of five independent experiments. Data were expressed as means ± SEM, and analyzed by two-way repeated measures ANOVA. **l** Dose responses of hPCMs towards known cardiotoxic anticancer therapeutics (doxorubicin, sorafenib, regorafenib, and ponatinib) upon cryopreservation. Forty-eight hours after drug treatment, cell viability was measured using CellTiter-Glo and normalized to DMSO vehicle control. *n* = 11 patients, each dose-response curve was plotted from six independent experiments. n.s. not sig*n*ificant (*P* > 0.05) paired two-tailed Student’s *t*-test

To further extend the application of the hPCM cell model, we sought to cryopreserve hPCMs. Cells were recovered at viability that was only ~10% lower than their matching freshly isolated cells (57.27% pre-freeze and 46.26% post-freeze, Fig. 5a, b). We achieved an average cell recovery rate of 66.74% (Fig. 5c), which was only a little below the average reported recovery rate for human stem cell-derived cardiomyocytes.^43^ Addition of Bleb into the cryopreservation media showed a trend towards improvement of post-thaw cell viability, which, however, did not reach statistical significance (Supplementary Fig. S6a). The viability of recovered cells was further evaluated in prolonged culture via live-cell imaging over a period of 6 days. Quantification of rod-shaped hPCMs revealed no significant differences between fresh and cryopreserved cells on either day 3 or day 6 (Fig. 5d). Next, to examine whether the freeze-thaw procedure caused cytoskeletal deformations, we performed immunofluorescence staining of cardiac markers ACTN2 and TNNT2. Cryopreserved cells demonstrated intact structure with clear striations, which was indistinguishable from freshly isolated cells, indicating preserved cell structure (Fig. 5e).

We further analyzed hPCMs by whole-cell patch clamping to assess their functional integrity. Baseline action potential parameters, including peak voltage, maximum upstroke velocity (dV/dt_max_), and action potential duration at 50 and 90% repolarization (APD50 and APD90), did not display any statistical differences before and after freezing (Fig. 5f). Interestingly, post-freeze hPCMs exhibited significantly lower resting membrane potential than freshly isolated ones (Fig. 5f). We further asked whether the response of cryopreserved cardiomyocytes to ion channel inhibitors was retained after freeze-thaw. To address this question, we recorded action potential tracings of the same single cells before and after drug treatment. As expected, sodium channel inhibitor TTX markedly reduced peak voltage and maximum upstroke velocity in cryopreserved cardiomyocytes, to the same extent as in freshly isolated ones (Fig. 5g and Supplementary Fig. S6b). In the same vein, calcium channel blocker nifedipine significantly reduced the duration of action potentials both before and after cryopreservation, evidenced by significant reductions in APD50 and APD90 (Fig. 5h and Supplementary Fig. S6c). We also measured cellular ATP levels, an indicator of mitochondrial integrity, and found that it, too, did not differ statistically with cryopreservation (*P* = 0.7745) (Fig. 5i).

To further corroborate the assessments of structure, electrophysiology, and metabolism, we performed RNA sequencing on four sets of paired pre- and post-freeze hPCMs. Surprisingly, not a single gene displayed significant differential expression (Fig. 5j), suggesting that the freeze-thaw cycle did not induce perturbations in global gene expression. Specifically, the gene expression of each of the functional modules (structural, metabolic, and ion channel) did not exhibit any statistical differences, which was further validated by real-time quantitative PCR (qPCR) (Fig. 5k). Collectively, these data indicated that cryopreservation minimally compromised the cellular, molecular and functional integrity of hPCMs, and suggest that the cryopreservation technique may be exploited for in vitro experiments, such as evaluation of drug effects.

To test this idea, we examined dose responses of four known cardiotoxic drugs,^44^ including doxorubicin, a chemotherapeutic agent, as well as three targeted anticancer therapeutics, including sorafenib, ponatinib, and regorafenib. None of the tested drugs exhibited differences in their IC_50_ values between fresh and cryopreserved cells (Fig. 5l), suggesting the possibility of using hPCMs for high-throughput and high-content screening.

### Cardiotoxicity screening of targeted therapies in hPCMs

**Fig. 6:**
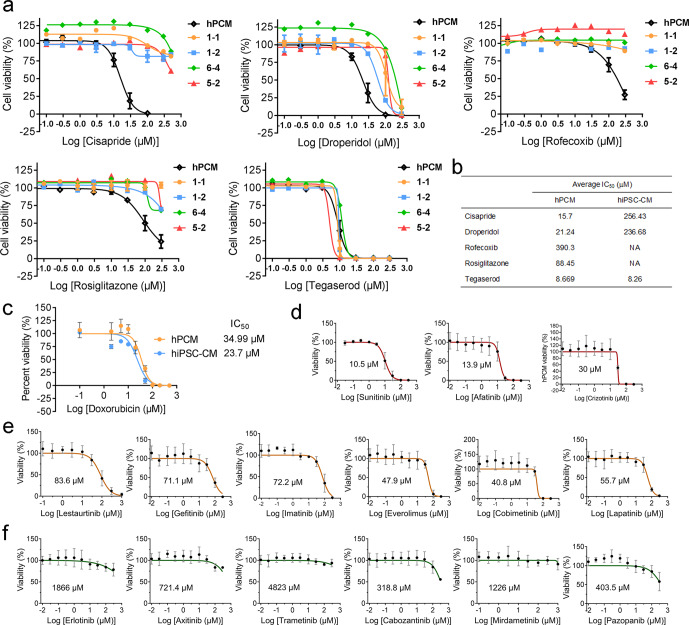
Cardiotoxicity screening of targeted therapies in hPCMs. **a** Dose-response curves based on cell viability assays of drugs in hPCMs (black line) and hiPSC-CMs (colored lines). Each drug was tested with hPCMs from five different patients, and hiPSC-CMs from four different hiPSC clones (1-1, 1-2, 6-4, 5-2). **b** IC_50_ values of drugs in hPCMs or hiPSC-CMs. NA represents cases where an IC_50_ could not be determined. **c** Doxorubicin cytotoxicity in hPCMs and hiPSC-CMs from the same patient. **d**–**f** Cardiotoxicity screening of 15 kinase inhibitors of different toxicity levels: high toxicity (sunitinib, afatinib, and crizotinib) (**d**), medium toxicity (lestaurtinib, gefitinib, imatinib, everolimus, cobimetinib, and lapatinib) (**e**), and low toxicity (erlotinib, axitinib, trametinib, cabozantinib, mirdametinib, and pazopanib) (**f**). *n* = 5 biological repeats for each drug

Cardiotoxicity is one of the major reasons for drug attrition during development and market withdrawal due to adverse cardiovascular events.^45^ To facilitate the detection of cardiac toxicity early in the drug discovery process, hiPSC-CMs are increasingly used as an in vitro platform for preclinical safety assessment.^13,46–48^ To test the potential of hPCMs in predicting cardiotoxicity, we selected five drugs of multiple categories that had been withdrawn from the market due to cardiac side effects. We measured the dose responses of these drugs both in hPCMs and hiPSC-CMs. Cisapride, droperidol, rofecoxib, and rosiglitazone all exhibited markedly lower IC_50_ values in hPCMs than in hiPSC-CMs (Fig. 6a, b), indicating that hPCMs may be more sensitive to direct cardiomyocyte toxicity. By contrast, tegaserod displayed comparable toxicity in both cell models, ruling out systematic deviation of drug sensitivity. Additionally, we used doxorubicin to evaluate the dose responses of hPCMs and hiPSC-CMs from the same patient. hPCMs had an IC_50_ value of 34.99 μM, while hiPSC-CMs exhibited an IC_50_ value of 23.7 μM, suggesting that primary and chemically induced cardiomyocytes may respond differently to the same drug (Fig. 6c).

Targeted therapeutics of signal transduction pathways used to treat cancer comprise a large group of drugs with significant cardiac toxicities.^49,50^ To further validate the use of hPCMs as a model for the prediction of drug-induced cardiomyocyte toxicity, we used an additional panel of kinase inhibitors with known profiles of toxicity. Sunitinib, afatinib, and crizotinib comprise a group of inhibitors with high-level cardiotoxicity. The multitargeted tyrosine kinase inhibitor sunitinib is widely recognized as a cardiotoxic drug, with evidence of congestive heart failure, electrocardiographic abnormalities, and hypertension.^51,52^ Its LD_50_ value in hiPSC-CMs was reported to be 12.70 μM.^44^ Epidermal growth factor receptor (EGFR) inhibitor afatinib was associated with an LD_50_ of 12.30 μM in hiPSC-CMs,^44^ but exhibits low frequencies of cardiac toxicity in the clinic.^53^ Anaplastic lymphoma kinase (ALK) inhibitor crizotinib is a drug that is generally well-tolerated in the clinic and can lead to Q-wave T-wave interval prolongation and bradycardia.^54^ In hiPSC-CMs, its effect on cell viability seemed very pronounced, with reported IC_50_ values of lower than 10 μM.^44,55^ The measured IC_50_ values of sunitinib, afatinib, and crizotinib in hPCMs were 10.5, 13.9, and 30 μM, respectively (Fig. 6d). The first two were very similar to reported values in hiPSC-CMs, while the latter was relatively higher, which was more concordant with it actual clinical profile. Overall, the IC_50_ values in the high-toxicity group did not exceed 30 μM. The medium-toxicity drug group consisted of lestaurtinib,^56^ gefitinib,^57^ imatinib,^44,50,56^ everolimus,^56^ cobimetinib,^58^ and lapatinib,^56,59^ all of which had been shown to show some level of toxicity. Accordingly, the IC_50_ values of these inhibitors remained in the range of 30–100 μM (Fig. 6e). Finally, erlotinib, axitinib, trametinib, cabozantinib, mirdametinib, and pazopanib were experimentally and clinically proven to be cardiac safe.^44,50,56^ Consistently, these drugs all had very large IC_50_ values (well above 100 μM) (Fig. 6f). Together, these results imply that hPCMs may be a reliable model for assessing cardiac toxicity in drug development.

### Population and single-cell transcriptomic responses of hPCMs to drugs

**Fig. 7:**
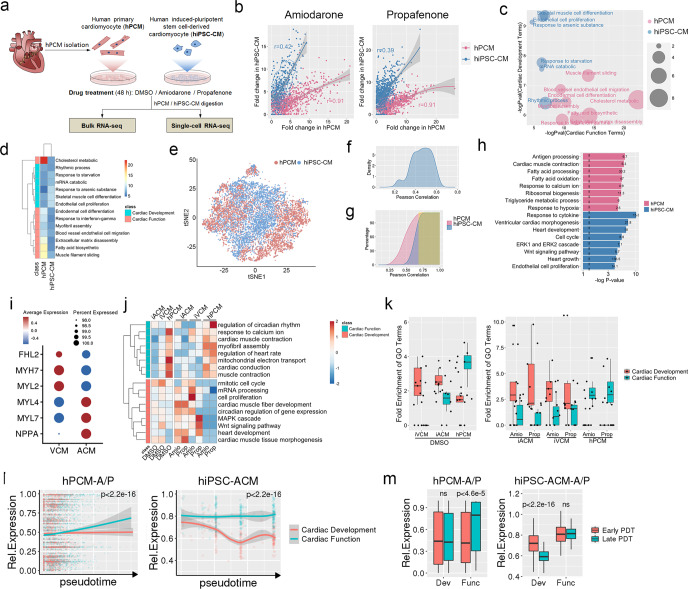
hPCMs display significant transcriptomic signatures of cardiac function in response to amiodarone and propafenone. **a** Schematic of study design. Human primary cardiomyocytes (hPCMs) from five donors and human-induced pluripotent stem cell-derived cardiomyocytes (hiPSC-CMs) from three individuals were treated with indicated drugs for 48 h, and their gene expression changes were detected by both bulk and single-cell RNA sequencing (scRNA-seq). **b** The transcriptome responses to amiodarone (top) or propafenone (bottom) in hPCMs or hiPSC-CMs, compared to their basal levels (DMSO). The density plots show the fold changes of differentially expressed genes (DEGs) in response to drugs, overlapped with base-level (DMSO) hPCM- or hiPSC-CM-specific genes. **c** Gene ontology (GO) analysis of the DEGs from (**b**). Selected top enrichment terms of overlapping DEGs between amiodarone and propafenone treatment in hPCMs (red) and hiPSC-CMs (blue) are shown. **d** Heatmap showing hierarchical clustering of the GO terms from (**c**) by fold enrichment. **e** *t*-distributed stochastic neighbor embedding (*tSNE*) visualization of 10,478 single cells from cultured hPCMs (red) and hiPSC-CMs (blue) treated with DMSO, amiodarone, or propafenone. **f** The distribution of Pearson correlations of the single-cell transcriptomes between hPCMs and hiPSC-CMs. **g** The stacks of Pearson correlations from F, showing cell-cell similarities of hPCMs and hiPSC-CMs, respectively. *R*^2^ > 0.6 was considered a high similarity (highlighted in yellow). **h** GO analysis of overlapping DEGs between amiodarone and propafenone treatment in hPCMs (red) and hiPSC-CMs (blue). The hPCMs and hiPSC-CMs were extracted from G with a cutoff of *R*^2^ > 0.6. **i** Dotplot showing the expression levels of atrial and ventricular marker genes of VCMs and ACMs, and their respective fractions. **j**, **k** Heatmap showing the *z*-scores (**j**) and boxplots (**k**) showing the fold enrichment of selected top GO terms of the DEGs in response to amiodarone (Amio) or propafenone (Prop) treatments in hiPSC-ACMs (i-ACM), VCMs (i-VCM) and hPCMs. **l** hPCMs (left) or hiPSC-CMs (right) ordered by pseudotime in response to amiodarone and propafenone treatment. Smoothed lines indicate the expression of cardiac function- or development-related genes along the trajectories upon drug treatment. **m** Boxplots showing the expression levels of cardiac function- or development-related genes in early or late pseudotime of hPCMs (left) or hiPSC-CMs (right), respectively. Early PDT: the first 10% cells in the pseudotime axis from N; Late PDT: the last 10% cells in the pseudotime axis from N. Dev: cardiac development-related genes; Func: cardiac function-related genes

To inquire the differences in the drug responses of hPCMs and hiPSC-CMs in greater depth and detail, we set out to perform bulk RNA sequencing of cells treated with either DMSO (D) control, amiodarone (A), or propafenone (P) for 48 h (Fig. 7a). hPCMs were isolated from the left atrial appendages of five patients undergoing coronary artery bypass graft surgery, while hiPSC-CMs were obtained from three healthy individuals using an undirected differentiation protocol. Cardioactive drugs of the same category (i.e., antiarrhythmics) were selected to observe both on-target (efficacy) and off-target (toxicity) effects and to deduce possible shared mechanisms of cellular response owing to commonalities in drug action. We first analyzed differentially expressed genes between DMSO-treated hPCMs and hiPSC-CMs (D-DEGs, Data S1). A total of 2484 were upregulated in hPCMs (hPCM-specific genes, red), while 6606 were more highly expressed in hiPSC-CMs (hiPSC-CM-specific genes, blue) (Supplementary Fig. S7a). According to Gene Ontology (GO) analysis, hPCM-specific genes showed significant enrichment for processes related to cardiac functions in mature cardiomyocytes, including ribosome biogenesis, lipid metabolism, as well as circadian rhythm (Supplementary Fig. S7b). By contrast, hiPSC-CMs, demonstrated strong enrichment in mitotic events, marked by high expression of cyclins (e.g., *CCNA2*, *CCNB1*, *CCND2*, *CCND3*, *CCNE2*, *CCNF*, etc), centromere genes (*CENPF*, *CENPX*, *CENPA*, *CENPT*, and *CENPW*), and genes relevant to spindle assembly (*PLK1*, *NUSAP1*, *AURKA*, and *AURKB*) (Supplementary Fig. S7c and Data S1). In addition, hiPSC-CMs also abundantly expressed genes involved in glycolysis (e.g., *ENO1/2*, *PFKFB2*, *PFKP*, *HK1*, and *PGAM1*), a known trait of immature cardiomyocytes (Supplementary Fig. S7c and Data S1). These results demonstrated that the two cardiomyocyte models each exhibited its unique molecular signature at a base level.

Next, we analyzed their respective drug-induced DEGs (i.e., A-DEG and P-DEG). A-DEG and P-DEG were compared against D-DEG to reveal cell type-specific fold changes in gene expression (Fig. 7b). Interestingly, for both amiodarone and propafenone treatment the correlation between gene fold changes displayed the same trend: high correlation for hPCM-specific genes (*r* = 0.91), and lower correlation for hiPSC-CM-specific genes (*r* = 0.42 and 0.39, for amiodarone and propafenone, respectively) (Fig. 7c). This indicates proportional changes in hPCM-specific genes in both models, suggesting that these genes may represent common traits of cardiomyocytes. On the contrary, a low correlation implies that hiPSC-CM-specific genes may be more representative of this specific model, lacking shared responses with other cardiomyocyte models. To further test this hypothesis, we performed a Gene Ontology (GO) analysis of treatment-induced DEGs in both models. Consistent with our supposition, hPCM response genes were highly enriched in terms related to cardiac function, whereas hiPSC-CM response genes were associated with cardiac development, evidenced by lower *P* values (Fig. 7c), as well as higher fold enrichment values (Fig. 7d).

Given that the hiPSC-CMs are usually a mixture of multiple subtypes, with their relative proportions varying by hiPSC cell source and differentiation protocols, and that the hPCMs used in our study were atrial cardiomyocytes, we took advantage of single-cell transcriptome profiling to ascertain whether the observed differences between hPCMs and hiPSC-CMs were due to their transcriptomic identities or to cellular heterogeneity. To this end, we applied single-cell RNA sequencing (scRNA-seq) to inquire single-cell transcriptomic responses of hPCMs and hiPSC-CMs to drug treatments (Fig. 7a). *t*-distributed stochastic neighbor embedding (*t*SNE) visualization of 6319 hPCMs and 4159 hiPSC-CMs that passed quality control filters uncovered substantial heterogeneity both between and within the two cardiomyocyte models (Fig. 7e and Supplementary Fig. S7e, f). To preclude the influence of cellular heterogeneity on drug-induced response, we performed pairwise cell-cell comparisons between hPCMs and hiPSC-CMs (Fig. 7f), and extracted the subset of cells with correlation coefficient *R*^2^ > 0.6 (Pearson correlation) for further analyses (Fig. 7g). In line with bulk RNA-seq data, single-cell drug-induced responses of hPCMs and hiPSC-CMs were associated with cardiac function and cardiac development, respectively (Fig. 7h and Data S2).

To better understand the roles of cell subtypes and heterogeneity in drug responses, we clustered single-cell data and separated them into 14 distinct populations based on their transcriptomic differences, each of which comprised cells from multiple donors (Supplementary Fig. S7g–i). Further, we performed correlation analysis on the signature genes of these 14 populations to narrow down the cluster-specific functions and grouped them into seven distinct K-clusters (K1–7) (Supplementary Fig. S7j), suggesting that some of the original populations share similar functions in response to drugs. In addition, the K-clusters were also clearly defined in the *t*SNE-embedded space, further confirming the significance of our clustering approach (Supplementary Fig. S7k). Inspection of the sample compositions of each K-cluster demonstrated that while K1 and K3 comprised cells from all treatment groups, other clusters were markedly skewed in their sample representation. For example, K2 and K4 mainly consisted of hiPSC-CMs, whereas K5, K6, and K7 were predominantly made up of hPCMs (Supplementary Fig. S7l, m). GO analysis revealed biological implications for each of the K-clusters. For example, the hiPSC-CM-dominant cluster K4 exhibited terms related to cell cycle progression, while the hPCM-dominant cluster K6 were primarily related to cardiac muscle function (Supplementary Fig. S7n).

For a fair comparison between hiPSC-CMs and hPCMs, we sorted out CM subtypes within hiPSC-CMs, and stratified them by the expression of atrial (*NPPA*, *MYL7*, and *MYL4*) and ventricular (*MYH7*, *FHL2*, and *MYL2*) marker genes (Supplementary Fig. S8 and Fig. 7i). Specifically, cells with high levels of atrial marker expression and low levels of ventricular marker expression were identified as atrial-like hiPSC-CMs (ACMs), whereas cells with low levels of atrial marker expression and high levels of ventricular marker expression were identified as ventricular-like hiPSC-CMs (VCMs). Next, we compared the basal gene expression between ACMs, VCMs, and hPCMs. Analysis of differentially expressed genes (DEGs) revealed that, compared to either ACMs or VCMs, hPCMs were more enriched in processes related to cardiac function, while ACMs and VCMs demonstrated relatively stronger enrichment in cardiac development-related terms (Fig. 7j, k, left, DMSO and Data S3,S4). Likewise, the DEGs following drug treatment in hPCMs were strongly enriched in processes related to cardiac function compared to ACMs, while those in ACMs following treatment were more strongly enriched in developmental pathways (Fig. 7j, k, right and Data S3 and S4).

Finally, we constructed the pseudotime trajectories of hPCMs and ACMs in response to amiodarone or propafenone treatment, and mapped the expression levels of cardiac function- or development-associated genes that had been annotated in GO analysis. In the hPCMs that appeared to be further along the trajectory, toward the drug-treated stage, the expression of cardiac function-related genes were significantly elevated (*P* < 2.2e^−16^), while that of cardiac development-related genes were not significantly altered (Fig. 7l, left). In comparison, ACMs responded to drug treatment by significant downregulation of cardiac development-associated genes (*P* < 2.2e^−16^), whereas cardiac function genes remained unchanged (Fig. 7l, right). Further analysis of expression levels of cardiac function- or development-related genes in early or late pseudotime (i.e., the first and last 10% of cells along the trajectories, respectively) revealed similar trends (Fig. 7m).

Taken together, we provided evidence that hPCMs and hiPSC-CMs respond differently to drug treatment, with the former preferentially responding with changes in genes related to cardiac function, and the latter exhibiting signatures of cardiac development.

## Discussion

Animal models, particularly rodents, have been used for many decades as the standard model in cardiac research, despite undeniable species differences in many aspects. With the advent of induced pluripotent stem cell technology, human somatic cell-derived cardiomyocytes have gained popularity due to its elimination of species differences and the possibility of performing large-scale in vitro experiments.^60^ Nowadays, the drug-induced pro-arrhythmic risk is frequently evaluated by in silico modeling or by using stem cell-derived cardiomyocytes,^61^ the latter of which exhibit an immature phenotype, and can sometimes generate false-positive predictions of Torsades de Pointe (TdP) risks.^62^ Adult hPCMs, by contrast, possess all native physiological and pharmacological properties and therefore can overcome many of the inherent limitations of current models.^18^ The use of human primary cells and tissue to bridge the translation gaps in cardiac drug discovery and safety assessment is a burgeoning field of research.^20,21,62,63^ Studies have reported the use of isolated human ventricular cardiomyocytes to simultaneously predict drug-induced inotropic and pro-arrhythmia risks,^21^ and to perform multiparametric profiling of ionotropic drugs.^20^

The pain point in the use of adult hPCMs for cardiac research is its notorious susceptibility to biochemical and mechanical perturbations. To confer biochemical protection, we replaced BDM with Bleb, and revealed superior protection with the latter, increasing the viability of cells to nearly threefold, an effect that was very specific to Bleb. Importantly, this method could be successfully applied to ventricular cardiomyocytes, both healthy and severely diseased, suggesting the possibility of this method to establish versatile hPCM models reflective of different cardiac compartments and different disease conditions.

Aside from isolation, adult hPCM culture is another technical challenge. We experimentally tested methods from sporadic reports of hPCM culture^23,24^ (data not shown) but failed to achieve the expected results. Inspired by our isolation results, we tested the use of Bleb in culture, and found that it was capable of retaining hPCM morphology, and surprisingly, metabolism. As a proof of concept, we showed that this model was also amenable to genetic manipulations, such as adenovirus-mediated gene expression. The ability to culture hPCMs that retain physiological characteristics for a period of time allows for the study of cardiac physiology and pharmacology, for which acute manipulations barely suffice.

To increase the flexibility of cell usage and to scale up experiments, hPCMs are ideally cryopreserved. However, cryopreservation of adult hPCMs is a daunting task due to the fragility of these cells. Cryopreservation of stem cell-derived cardiomyocytes is relatively well established,^64^ whereas, with respect to primary cardiomyocytes, it has been only attempted in neonatal rats.^65^ To our knowledge, no successful report of post-thaw recovery of cells with such complex and rigid cellular structure exists. Leveraging the high quality of our isolated hPCMs, we successfully developed a strategy to recover hPCMs from liquid nitrogen. More importantly, we showed that post-thaw cells are compatible with cell culture, gene expression analysis, electrophysiological analysis, metabolic analysis, and even pharmacological evaluation, indicating the potential application of this cellular model for large-scale, high-throughput drug discovery processes.

Cardiac safety testing is currently accomplished using animal models, heterologous expression systems and stem cell-derived cardiomyocytes. Most efforts are directed at predicting pro-arrhythmic effects of drugs, whereas direct cardiomyocyte toxicity, such as mitochondrial toxicity, may evade screening. Although the majority of cardiac adverse events are attributed to arrhythmia, the possibility of other toxic effects on cardiomyocytes cannot be excluded. Indeed, without measuring electrophysiology, we observed apparent toxicity with 5-hydroxytryptamine receptor 4 (5-HT4) agonists cisapride and tegaserod. Interestingly, while the latter exhibited equal cytotoxicity in both model systems, cisapride displayed much greater toxicity in primary cells, which is unlikely explained by previous studies supporting human ether-a-go-go-related gene (hERG) channels as the major contributor to cisapride cardiotoxicity.^66^ In addition to cisapride, dopamine D2 receptor antagonist droperidol, cyclooxygenase-2 (COX-2) inhibitor rofecoxib, and peroxisome proliferator-activated receptor γ (PPARγ) agonist rosiglitazone, all displayed greater toxicity in hPCMs compared to hiPSC-CMs. This suggests that these specific pathways or relevant off-target pathways are more important to the viability of hPCMs than hiPSC-CMs. Interestingly, doxorubicin exhibited relatively greater toxicity in hiPSC-CMs. Doxorubicin is a chemotherapeutic drug that acts by forestalling DNA replication as a means of suppressing tumor growth. Therefore, this drug might have a more pronounced effect in cells with proliferative potentials, such as in hiPSC-CMs, while exerting less impact on terminally differentiated, non-proliferative hPCMs. The detailed mechanisms by which these two models differ in predicting drug toxicity certainly remain to be explored in future studies, and the knowledge gained will be crucial to stratifying types of cardiotoxicity.

By comparing the transcriptomic responses of hPCMs and hiPSC-CMs to cardioactive drugs, we unraveled their unique response patterns. hPCMs exhibited a response gene signature that reflected cardiac functions, while hiPSC-CMs displayed developmental enrichment of drug response. Although these two models are known to be inherently different, in terms of developmental stage and cardiomyocyte subtype, we showed that even for the cell subset that showed high transcriptome similarity, the drug-induced response patterns were still evident. Of note, in our second approach, when we interrogated individual cell clusters, two mixed clusters were present (K1 and K3), yet their GO term fold enrichment values were markedly different between their hPCM and hiPSC-CM components (Fig. 7m). These data unanimously highlight hPCMs as a cardiomyocyte model that faithfully recapitulates attributes of mature cardiomyocytes and their functional properties. It will be interesting to explore the possibility to apply hPCMs isolated from different cardiac compartments or individuals for personalized pharmacological evaluation.

In summary, we developed and carefully evaluated a methodology for the isolation, culture, and cryopreservation of hPCMs. Our transcriptome analyses of drug response at both population and single-cell levels unveiled hPCMs as a potentially desirable cellular model for the study of cardiac physiology, drug discovery, and safety evaluation.

## Materials and methods

### Human samples

All hPCMs used in this study were isolated either from the left atrial appendages of patients undergoing coronary artery bypass graft, aortic valve replacement, mitral valve replacement or mitral valvuloplasty surgeries, or from the ventricles of patients undergoing ventricular aneurysmectomy, transaortic septal myectomy, or heart transplantation. This study included a total of 136 patients (101 male and 35 female), mean age of 57.7 ± 11.3 years (median 60 years) (Please see Supplementary Methods for detailed patient information). Written informed consent was obtained from all patients. The study was approved by the Ethics Committee of Fuwai Hospital, Chinese Academy of Medical Sciences, and Peking Union Medical University, and conducted according to the Declaration of Helsinki. For all studies concerning hPCM cryopreservation, we used paired pre- and post-freeze hPCM samples (i.e., from the same patient), to preclude the influence of patient variability on data interpretation.

### Isolation and culture of human cardiomyocytes

Cardiac specimens were transported in ice-cold cardioplegic solution (University of Wisconsin solution, UW; Belzer, CHD120419) to a microtome (Leica Biosystems, VT1200S) for sectioning into 300-μm-thick tissue slices, which were further minced into smaller fragments. Minced tissues were transferred to a Tyrode’s solution (126 mM NaCl, 4.4 mM KCl, 5 mM MgCl2·6H2O, 5 mM NaH_2_PO_4_, 5 mM HEPES, 22 mM glucose, 20 mM taurine, 5 mM creatine, 5 mM sodium pyruvate, supplemented with either 10 µM Bleb, or 20 mM BDM) containing 250 U/ml collagenase type II and 1.2 U/ml protease XXIV (Sigma) for digestion at 37 °C with gentle agitation in a shaking water bath until the supernatant turned visibly cloudy, and significant amounts of rod-shaped cardiomyocytes were observed under a light microscope. The supernatant was discarded at this step. The tissue was then transferred to a fresh digestion solution but without protease. Cardiomyocytes were collected by gentle centrifugation (100×*g*, 3 min, 4 °C) of the supernatant, while the remaining tissue was immersed in the fresh enzymatic solution for further digestion. This cycle of digestion collection was repeated several times until the residual tissue turned pale and yielded no more cardiomyocytes. For electrophysiological and RNA-seq studies, calcium concentrations were gradually restored to 1.8 mM.

Isolated cardiomyocytes were plated onto 200 μg/ml laminin-coated surfaces, and maintained in MEM (Gibco, 42360099) supplemented with 10% FBS (Gibco), 100 U/ml penicillin-streptomycin (Gibco), 100 μg/ml Primocin (InvivoGen), and 10 µM Bleb. For all experiments comparing the effects of BDM versus Bleb, BDM was used at a concentration of 20 mM.

### Human-induced pluripotent stem cell-derived cardiomyocytes (hiPSC-CMs)

hiPSC-CMs used for bulk and single-cell RNA-seq experiments were purchased from Cellapy Biological (Beijing, China), including drug screening-grade hiPSC-CMs (Cat# CA2201106, Lot# F2718G2716, female, 55 years, skin), and two types of research-grade hiPSC-CMs (Cat# CA2204106, Lot# I17E220, female, 28 years, urine; Cat# CA2101106, Lot# WFJH3116IV161, male, 34 years, urine). These cells were generated and purified using published protocols,^67,68^ and used on differentiation day 45 in our study.

hiPSC-CMs used in drug response assays (Patient IDs 1-1/2, 6-4, and 5-2) were produced in-house using the same undirected differentiation protocol.^67^ Briefly, the patient skin tissue was digested using Dispase (Gibco, 17105-041) at 4 °C overnight. The epidermis was removed, and the dermis was chopped into small pieces, and transferred into six-well plates precoated with 0.1% gelatin (Sigma-Aldrich, G7041-100g). A glass coverslip was mounted on top and a fresh F-12 medium (Gibco, 11330057) was added. The medium was changed every 2–3 days, and human fibroblasts could be harvested after 1–2 weeks. pCXLE-hOCT3/4-shp53, pCXLE-hSK, and pCXLE-hUL were transfected into human primary fibroblasts by electroporation using a Human Dermal Fibroblasts Nucleofector^®^ Kit (Lonza, VPD-1001). Transfected cells were plated into six-well plates coated with recombinant human vitronectin (rh-vitronectin), and cultured using a chemically defined medium (hPSC-CDM^TM^, Nanjing Boshou Biotech, 400105) supplemented with hydrocortisone (Sigma, H0888-10g) before reaching 20% confluency. The medium was then switched to hPSC-CDM, and changed every day until hiPSC-like clones appear. Clones were picked and transferred to a well of a 24-well plate coated with rh-vitronectin. hPSC-CDM supplemented with 10 μM Y27632 (Stemcell Technologies, 72307) was used for the first day of culture until cells were attached to the bottom of the well. hPSC-CDM without supplementation was used to support the growth of hiPSC clones. hiPSC cells were passaged and expanded into 12-well-plates. Cardiac differentiation began when cells reached 90–100% confluency. RPMI 1640 (Gibco, 11875093) with 1 × B27™ supplement, minus insulin (Gibco, A1895601) was used as the basal medium. During cardiac differentiation 3 μM CHIR-99021 (Selleck, S2924) and 5 μM IWR-1(Selleck, S7086) was added when changing the medium on day 0 and day 3, respectively. The medium was changed to back to RPMI 1640 with 1 × B27™ Supplement, minus insulin on day 5. On day8 of differentiation, the medium was switched to RPMI 1640 with B27™ supplement (1×), serum-free (Gibco, 17504044), and refreshed on day 11. On day14, the medium was switched to DMEM without glucose (Gibco, 11966025), but supplemented with l-Lactic acid (Kako Chemicals, 129-02666), for purification for 2–3 days. Following replating, the medium was changed every day (RPMI 1640 with B27™ supplement (1×), serum-free) until use. hiPSC-CMs derived from clones 1-1, 1-2, and 5-2 were all used between 31 and 37 days, while 6-4 was used at day 65 to determine the influence of differentiation day on drug response.

### RNA sample preparation and RNA-seq

hPCMs were pelleted and flash-frozen at −80 °C for storage. Total RNA was isolated using GeneJET RNA Purification Kit (Thermo Scientific, K0731) for downstream gene expression analysis. The quantity and quality of RNA were verified by NanoDrop 2000 (Thermo Scientific). All RNA samples with an equivalent starting amount (500 ng/sample) were operated to construct mRNA-Seq libraries using KAPA mRNA HyperPrep Kit (Illumina, KK8581), in accordance with the manufacturer’s instructions. Each synthesized DNA library was uniquely indexed, allowing all samples to pool together. The size and concentration of the synthesized DNA libraries were measured using 2100 Bioanalyzer (Agilent) and Qubit (Thermo Fisher Scientific) respectively. Libraries were diluted to 2–3 pM and then sequenced on Illumina NextSeq 500 platform using the 75 cycles of the NextSeq 500/550 High Output Kit v2.5 kit (Illumina, 20024906). Raw data were collected by sequencing and stored in fastq format.

### RNA-seq analysis

Reads were mapped to the human reference genome GRCh38/hg38 using subread (v.1.6.2). Uniq reads were kept and then assigned to Rsubread’s in-built RefSeq gene annotations using the “featureCounts” function (R v.1.30.9).^69^ For gene filtering, only ones with RPKM values greater than 1 in at least two samples were retained for downstream analysis.^70^ Limma (v.3.36.5)^71^ was used to identify differentially expressed genes between CMs before and after calcium reintroduction, cryopreservation, as well as those between Langendorff- and TSAD-isolated cells. Adjusted *P* values are used to overcome the multiple testing problem. Genes with log2FC (i.e., log_2_(fold-change value)) over ± 0.58 and with adjusted *P* values (false discovery rate) <0.05 were taken as significantly differentially expressed genes.

Cell type-specific gene expression in myocardial samples was imputed in CIBERSORTx as described by ref. ^72^ The signature matrix file of the human left atrium was conducted using our earlier single-cell dataset,^73^ and the top 2000 genes were used to impute the cell fractions and gene expression in distinct cell types. Imputed CM gene expression was then used to calculate the Spearman correlation of different samples.

### Cryopreservation of hPCMs

Immediately following isolation and calcium reintroduction, one aliquot of hPCMs was directly subjected to experimentation, while another aliquot from the same donor was frozen using CryoStor^®^ CS10 following a standard protocol, and stored in liquid nitrogen until use. Briefly, hPCMs were gently pelleted and resuspended in a cryopreservation medium at a concentration of 1 × 10^6^ cells/ml. Cryovials were transferred to a CoolCell™ container for slow cooling (−1 °C per minute). Thawing of cells was achieved through gentle swirling in a 37 °C water bath and dropwise addition of culture media (ten times the volume of cryopreservation medium). Cells were gently pelleted and directly used for subsequent experiments.

### Electrophysiology

Membrane currents and action potentials (APs) were stimulated and recorded by the whole-cell patch-clamp technique. All chemicals were purchased from Sigma-Aldrich unless otherwise indicated. Prior to the experiment, CMs were seeded and cultured for a minimum of 2 h on laminin-coated glass coverslips (diameter 12 mm) in 24-well plates. Glass coverslips with CMs were transferred into the cell chamber on an inverted microscope (IX71, Olympus), which were superfused at 1.5 ml/min with an extracellular solution at room temperature. Only quiescent rod-shaped cells showing clear cross striations were selected for recording. Glass pipettes with tip resistances of 3–4 MΩ were pulled using borosilicate glass capillary tubes (BF150-86-10, Sutter Instruments) with a micropipette puller (P97, Sutter Instruments) and filled with the appropriate internal solution. The cell membrane was ruptured by gentle suction to establish a whole-cell configuration after a giga-ohm seal was obtained. Membrane currents were recorded in voltage-clamp mode, and action potentials were recorded in current-clamp mode. Data were acquired and sampled at a rate of 100 kHz rate for *I*_Na_ and *I*_Ca,L_, and 20 kHz for *I*_to_ and APs, with the EPC-10 patch-clamp amplifier (HEKA) using PatchMaster software (HEKA). Detailed methods for the recording of action potentials and membrane currents are included in the Supplementary Materials.

### Single-cell RNA sequencing

Single-cell sequencing libraries were generated using the ICELL8 platform (Takara Bio USA) following the manufacturer’s instructions. Briefly, isolated hPCMs or digested hiPSC-CMs were stained with a mixture of Hoechst 33342 and propidium iodide, washed with PBS, and counted on a Moxi^TM^ Automated Cell Counter. A cell suspension of 20,000 cells/ml was subjected to the MultiSample NanoDispenser (MSND, Wafergen Biosystems) for single-cell preparation. The dispensed cells were then imaged using the Imaging Station, and single, live cells (Hoechst-positive, propidium iodide-negative) were selected for reverse transcription and first-step amplification in a Chip Cycler (Bio-Rad). The resulting cDNA was purified and size-selected with Agencourt AMPure XP beads (Beckman Coulter, A63880). One ng of purified cDNA was applied to generate a sequencing library using Nextera XT DNA sample preparation kit (Illumina). Libraries were sequenced on the NextSeq 500 sequencer (Illumina) using the 26 nt and 50 nt paired-end sequencing protocol.

### Single-cell RNA-seq analyses

The *FindAllMarkers* and *FindMarkers* were utilized to identify differentially expressed genes with parameters *test.use* = *Wilcox, min.pct* = *0.25, thresh.use* = *0.25, only.positive* = *TRUE* for clarifying the signature genes of each cell group, and FALSE for identifying the differential expression genes between multiple cell groups. The adjusted *P* value <0.05 of the genes were considered significant DEGs.

Gene ontology analysis was done with the DAVID database. KEGG pathways and biological processes were carried out using Fisher’s exact test and corrected by FDR 5%. The terms specifically enriched in hPCMs and associated with mature cardiomyocytes functions were defined as cardiac functions terms, while the hiPSC-CM-specific terms were defined as cardiac development terms.

Processed *Seurat* object was converted to *SingleCellExperiment* format and imported to *Monocle3* and followed by cell clustering and *UMAP* visualization. The cells were then separated by cell models which contain both non-treated and amiodarone or propafenone treated groups. For each subset, a nonlinear model was used to interpret the topology-preserving single-cell embeddings and developed the trajectories. The pseudotime coordinates of each cell were then extracted from *Monocle3* and were matched with their expression matrix to compute the expression levels of the gene in cardiac functions or development terms. The first or last 10% of cells in the pseudotime axis were defined as an early or late stage of drug treatment, and the expression levels of cardiac function or development-related genes were directly compared between them, respectively. Median values were presented with standard deviation.

### Correlation analysis

For cell-cell pairwise transcriptome correlations analysis, we extracted the common genes in each cell types and calculated their Pearson correlations based on log-normalized expression levels. For cell functional clustering, we identified differentially expressed genes and calculated their average expression levels. The Spearman correlation and the hierarchical clustering based on Euclidean distance were then performed with those average values. The complete method was used in Pearson or Spearman correlations.

### Statistical analysis

For gene enrichment analyses, we used the cumulative binomial distribution test. For overlapping, we used Fisher’s exact test. For comparisons between two groups of equal sample size (and assuming equal variance), an unpaired two-tailed Student’s *t*-test was performed or in cases of unequal sample sizes or variance, Welch’s unequal variances *t*-test was performed, as indicated. For multiple comparison testing, a one-way analysis of variance (ANOVA) accompanied by Tukey’s post hoc test was used as appropriate. Paired *t*-tests and repeated measures ANOVA were applied to paired data when applicable. Differentially expressed genes in the scRNA-Seq data were identified using a Wilcoxon rank-sum test. Data were expressed as means ± SEM or mean ± SD, as indicated in the figure legends. *P < 0.05, **P < 0.01, ***P < 0.001, n.s. not significant. *P* < 0.05 was considered statistically significant.

## Supplementary information

Supplementary Materials

Dataset 1

Dataset 2

Dataset 3

Dataset 4

## Data Availability

All data are available in the main text or Supplementary Materials.
