# Supplementary material for: Functional isolation, culture and cryopreservation of adult human primary cardiomyocytes
Source: Signal Transduct Target Ther. 2022 Jul 27;7:254. doi: 10.1038/s41392-022-01044-5 (PMC9325714; doi:10.1038/s41392-022-01044-5)
Supplement: Supplementary file 1 — Supplementary Materials [file 41392_2022_1044_MOESM1_ESM.docx]

Supplementary Materials for

Functional isolation, culture and cryopreservation of adult human primary cardiomyocytes

Bingying Zhou, Xun Shi, Xiaoli Tang, Quanyi Zhao, Le Wang, Fang Yao, Yongfeng Hou, Xianqiang Wang, Wei Feng, Liqing Wang, Xiaogang Sun, Li Wang, Shengshou Hu

Correspondence to: huss@fuwai.pumc.edu.cn

**This PDF file includes:**

Materials and Methods

Figures. S1 to S8

**Other Supplementary Materials for this manuscript include the following:**

Data S1 to S4

DEGs between hPCMs and hiPSC-CMs from buk RNA-seq

GO analysis of DEGs between hPCMs and hiPSC-CMs from scRNA-seq

DEGs among ACMs, VCMs and hPCMs

GO enrichment of DEGs among ACMs, VCMs and hPCMs upon drug treatment

Materials and Methods

Patient information

Patient information, including age, sex, diagnosis, surgery type, and tissue source, are summarized below.

**Table 1.** Patient information.

| Patient ID | Age | Sex | Primary diagnosis | Surgery | Tissue source |
| --- | --- | --- | --- | --- | --- |
| 1 | 24 | F | valvular heart disease | MVP+MVR | LAA |
| 2 | 67 | M | coronary heart disease | CABG | LAA |
| 3 | 72 | M | valvular heart disease | AVR | LAA |
| 4 | 64 | F | coronary heart disease | CABG | LAA |
| 5 | 56 | F | coronary heart disease | CABG | LAA |
| 6 | 53 | M | coronary heart disease | CABG | LAA |
| 7 | 65 | M | valvular heart disease | MVP+CABG | LAA |
| 8 | 58 | M | coronary heart disease | CABG | LAA |
| 9 | 46 | M | coronary heart disease | CABG | LAA |
| 10 | 45 | M | coronary heart disease | CABG | LAA |
| 11 | 61 | M | coronary heart disease | CABG | LAA |
| 12 | 56 | M | coronary heart disease | CABG | LAA |
| 13 | 55 | F | coronary heart disease | CABG | LAA |
| 14 | 74 | F | coronary heart disease | CABG | LAA |
| 15 | 65 | F | coronary heart disease | CABG | LAA |
| 16 | 45 | M | coronary heart disease | CABG | LAA |
| 17 | 61 | F | valvular heart disease | AVR | LAA |
| 18 | 66 | M | coronary heart disease | CABG | LAA |
| 19 | 54 | M | coronary heart disease | CABG | LAA |
| 20 | 71 | M | coronary heart disease | CABG | LAA |
| 21 | 48 | M | coronary heart disease | CABG | LAA |
| 22 | 71 | M | coronary heart disease | CABG | LAA |
| 23 | 34 | F | valvular heart disease | MVP+MVR | LAA |
| 24 | 53 | M | coronary heart disease | CABG | LAA |
| 25 | 72 | M | senile degenerative heart valvular disease | AVR | LAA |
| 26 | 57 | M | coronary heart disease | CABG | LAA |
| 27 | 51 | M | coronary heart disease | CABG | LAA |
| 28 | 44 | M | hypertrophic obstructive cardiomyopathy | Transaortic septal myectomy | LAA |
| 29 | 73 | M | coronary heart disease | CABG | LAA |
| 30 | 56 | M | valvular heart disease | MVP+MVR | LAA |
| 31 | 66 | M | valvular heart disease | MVP+MVR | LAA |
| 32 | 67 | M | coronary heart disease | CABG+AVR+MVP | LAA |
| 33 | 51 | M | coronary heart disease | CABG | LAA |
| 34 | 71 | M | coronary heart disease | CABG | LAA |
| 35 | 61 | M | coronary heart disease | CABG+AVR | LAA |
| 36 | 52 | M | coronary heart disease | CABG | LAA |
| 37 | 75 | F | coronary heart disease | CABG | LAA |
| 38 | 63 | F | valvular heart disease | MVP+MVR | LAA |
| 39 | 40 | F | rheumatic heart disease | MVR+AVR | LAA |
| 40 | 61 | M | coronary heart disease | CABG | LAA |
| 41 | 56 | M | valvular heart disease | MVP | LAA |
| 42 | 69 | F | coronary heart disease | CABG | LAA |
| 43 | 72 | F | coronary heart disease | CABG | LAA |
| 44 | 60 | M | coronary heart disease | CABG+MVP+MVR | LAA |
| 45 | 52 | M | hypertrophic obstructive cardiomyopathy | MVP | LAA |
| 46 | 73 | F | coronary heart disease | CABG | LAA |
| 47 | 67 | M | coronary heart disease | CABG | LAA |
| 48 | 32 | M | coronary heart disease | CABG | LAA |
| 49 | 58 | M | coronary heart disease | CABG | LAA |
| 50 | 60 | M | coronary heart disease | CABG | LAA |
| 51 | 59 | F | valvular heart disease | CABG+MVP+MVR | LAA |
| 52 | 47 | M | non-rheumatic aortic valve insufficiency | AVR | LAA |
| 53 | 63 | M | coronary heart disease | CABG | LAA |
| 54 | 72 | M | coronary heart disease | CABG | LAA |
| 55 | 68 | M | coronary heart disease | CABG | LAA |
| 56 | 56 | M | coronary heart disease | CABG | LAA |
| 57 | 63 | M | coronary heart disease | CABG | LAA |
| 58 | 51 | M | coronary heart disease | CABG | LAA |
| 59 | 65 | M | valvular heart disease | MVP | LAA |
| 60 | 57 | M | valvular heart disease | AVR | LAA |
| 61 | 47 | F | coronary heart disease | CABG | LAA |
| 62 | 63 | F | coronary heart disease | CABG | LAA |
| 63 | 66 | M | coronary heart disease | CABG | LAA |
| 64 | 66 | F | coronary heart disease | CABG | LAA |
| 65 | 65 | M | coronary heart disease | CABG | LAA |
| 66 | 64 | M | coronary heart disease | CABG | LAA |
| 67 | 67 | M | coronary heart disease | CABG | LAA |
| 68 | 62 | M | valvular heart disease | MVP+MVR | LAA |
| 69 | 63 | F | valvular heart disease | CABG+MVP+MVR | LAA |
| 70 | 61 | M | coronary heart disease | CABG | LAA |
| 71 | 43 | M | valvular heart disease | MVP | LAA |
| 72 | 57 | M | coronary heart disease | CABG | LAA |
| 73 | 42 | M | coronary heart disease | CABG | LAA |
| 74 | 64 | M | coronary heart disease | CABG | LAA |
| 75 | 53 | M | coronary heart disease | CABG | LAA |
| 76 | 62 | M | valvular heart disease | AVR | LAA |
| 77 | 66 | M | coronary heart disease | CABG | LAA |
| 78 | 44 | M | coronary heart disease | CABG | LAA |
| 79 | 65 | M | coronary heart disease | CABG | LAA |
| 80 | 14 | M | coronary heart disease | AVR+MVP+MVR | LAA |
| 81 | 40 | F | valvular heart disease | MVR+TVP | LAA |
| 82 | 44 | F | coronary heart disease | CABG | LAA |
| 83 | 70 | F | coronary heart disease | CABG | LAA |
| 84 | 66 | M | coronary heart disease | CABG | LAA |
| 85 | 52 | M | coronary heart disease | CABG+MVP+MVR | LAA |
| 86 | 66 | F | coronary heart disease | CABG | LAA |
| 87 | 50 | M | coronary heart disease | CABG | LAA |
| 88 | 71 | M | coronary heart disease | CABG | LAA |
| 89 | 58 | M | coronary heart disease | CABG | LAA |
| 90 | 66 | F | coronary heart disease | CABG | LAA |
| 91 | 64 | M | coronary heart disease | CABG | LAA |
| 92 | 56 | M | coronary heart disease | CABG | LAA |
| 93 | 66 | F | coronary heart disease | CABG | LAA |
| 94 | 37 | F | infective endocarditis | MVP+MVR | LAA |
| 95 | 65 | F | coronary heart disease | CABG | LAA |
| 96 | 61 | M | coronary heart disease | CABG | LAA |
| 97 | 55 | M | coronary heart disease | CABG | LAA |
| 98 | 57 | M | coronary heart disease | CABG | LAA |
| 99 | 38 | M | valvular heart disease | MVP | LAA |
| 100 | 56 | F | hypertrophic obstructive cardiomyopathy | Transaortic septal myectomy+CABG | LAA |
| 101 | 22 | F | aneurysm of ascending aorta | AVR | LAA |
| 102 | 65 | M | coronary heart disease | CABG | LAA |
| 103 | 65 | M | coronary heart disease | CABG | LAA |
| 104 | 40 | M | coronary heart disease | CABG | LAA |
| 105 | 60 | F | valvular heart disease | MVP+TVP | LAA |
| 106 | 68 | F | coronary heart disease | CABG | LAA |
| 107 | 67 | M | valvular heart disease | AVR+CABG | LAA |
| 108 | 65 | M | coronary heart disease | AVR+CABG | LAA |
| 109 | 48 | F | valvular heart disease | MVR+MVP+AVR | LAA |
| 110 | 55 | M | valvular heart disease | AVR | LAA |
| 111 | 65 | M | coronary heart disease | CABG | LAA |
| 112 | 71 | M | coronary heart disease | CABG | LAA |
| 113 | 53 | M | coronary heart disease | CABG | LAA |
| 114 | 58 | M | coronary heart disease | CABG | LAA |
| 115 | 62 | M | coronary heart disease | CABG | LAA |
| 116 | 54 | M | coronary heart disease | CABG | LAA |
| 117 | 53 | M | coronary heart disease | CABG | LAA |
| 118 | 67 | M | coronary heart disease | CABG | LAA |
| 119 | 48 | M | coronary heart disease | CABG | LAA |
| 120 | 37 | M | congenital heart disease | Bentall’s procedure | LAA |
| 121 | 66 | M | coronary heart disease | CABG | LAA |
| 122 | 59 | M | coronary heart disease | Ventricular aneurysmectomy + CABG | LV |
| 123 | 32 | M | dilated cardiomyopathy | Orthotopic heart transplantation | LV |
| 124 | 68 | M | coronary heart disease | CABG | LAA |
| 125 | 73 | F | valvular heart disease | AVR | LAA |
| 126 | 61 | M | coronary heart disease | CABG | LAA |
| 127 | 54 | F | valvular heart disease | AVR+CABG | LAA |
| 128 | 50 | M | valvular heart disease | MVP | LAA |
| 129 | 73 | M | coronary heart disease | CABG | LAA |
| 130 | 59 | M | coronary heart disease | CABG | LAA |
| 131 | 54 | M | coronary heart disease | CABG | LAA |
| 132 | 51 | M | coronary heart disease | CABG | LAA |
| 133 | 56 | F | rheumatic heart disease | MVR+AVR | LAA |
| 134 | 71 | M | coronary heart disease | CABG | LAA |
| 135 | 49 | M | coronary heart disease | CABG | LAA |
| 136 | 55 | M | coronary heart disease | CABG | LAA |

Cell viability staining

Cell viability staining was performed using the LIVE/DEAD™ Viability/Cytotoxicity Kit (ThermoFisher, L3224), following the manufacturer’s instructions. Stained cells were imaged using a microscope (Leica, DMI4000B), and quantified by manual counting of green (live) versus red (dead) cells in ImageJ (v1.8.0). Cell viability was as follows: Cell viability = number of live cells/ total cell number × 100%. Viable cells were identified by 1) calcein-AM (green) staining, and 2) rod-shape morphology. Exclusion criteria include: 1) round cells with bright green fluorescence (apoptotic), and 2) apparent membrane blebbing or vacuolization.

Measurement of action potentials and membrane currents

AP recordings were performed using a bath solution containing (in mM) NaCl 140, KCl 3.5, CaCl_2_ 2, MgCl_2_ 1, HEPES 10, glucose 10, and NaH_2_PO_4_ 1.25, pH 7.4 with NaOH, and a pipette solution containing (in mM) NaCl 5, CaCl_2_ 0.1, Mg-ATP 2, HEPES 10, ATP 5, K-Gluconate 140, MgCl_2_ 1, and EGTA 1, pH 7.2 with KOH. Resting membrane potential was adjusted to -80 mV via the injection of a negative offset current before APs recording^44^. APs were elicited by injection of 1.1-1.2 times the threshold current pulses for 10 ms at a frequency of 1 Hz. Offline data analysis and statistical evaluations were performed with Clampfit 10.6 (Axon) and the following parameters of action potential were measured: resting membrane potential (RMP), action potential amplitude (APA), maximal rate of depolarization (V_max_) and action potential duration at 50 and 90% of repolarization (APD 50 and APD 90, respectively).

Membrane currents were measured using the following solutions. For the recording of *I*_Na_, the bath solution consisted of (in mM) Choline chloride 115, NaCl 20, CsCl 5.4, MgCl_2_·6H_2_O 1, CaCl_2_·2H_2_O 1.8, D-Glucose 10, HEPES 10, BaCl_2_ 0.3, CdCl_2_ 0.1, Nifedipine (Sigma, N7634) 0.01, pH 7.4 with TEAOH; the intracellular solution consisted of (in mM) CsCl 50, NaCl 10, HEPES 10, CsF 60, EGTA 20, pH 7.2 CsOH. For *I*_Ca,L_ measurements, the external solution was (in mM): Triethanolamine (TEA)-Cl 140, MgCl_2_·6H_2_O 2, CaCl_2_·2H_2_O 10, HEPES 10, D-Glucose 5, pH 7.4 with TEA-OH; the internal solution was (in mM): CsCl 120, MgCl_2_·6H_2_O 1, HEPES 10, EGTA 10, Na_2_-GTP 0.3, Mg-ATP 4, pH 7.2 CsOH. For *I*_to_ recordings, the external solution used had the following composition (in mM): NaCl 40, Choline chloride 100, KCl 3.5, HEPES 10, D-glucose 10, NaH_2_PO_4_ 2H_2_O 1.25, MgCl_2_ 1, CaCl_2_ 2, BaCl_2_ 0.1，Nifedipine 0.001, tetrodotoxin (TTX, Alomone Labs, T-550) 0.003, pH 7.4 with NaOH; the internal solution was (in mM): K-Gluconate 140, NaCl 5, CaCl_2_ 0.1, MgCl_2_ 1, HEPES 10, EGTA 1, Mg-ATP 2, pH 7.2 with KOH.

To evaluate the response of ion channels to specific inhibitors, we determined used TTX, nifedipine and 4-AP to inhibit voltage-gated sodium channels, L-type voltage-gated calcium channels, and voltage-gated poytassium channels, respectively. Control and increasing concentrations drug solutions were applied to the exterior of the cell in a cumulative manner via a 7-port gravity flow system at 0.3 mL/min. *I*_Na_, *I*_Ca,L_ and *I*_to_ were measured respectively during a train of 100, 300 and 500-ms step depolarizations to +10, +30 and +40 mV at 0.05 Hz in the presence of the agents. Cells were excluded from analysis if peak decreased irreversibly during the protocol, but it was not possible to measure currents at every concentration studied in each cell. The percentage of current peak amplitudes at different drug concentrations was compared with that of the vehicle control. To obtain IC_50_ values, the fractional blocks obtained at different drug concentrations were fitted iteratively (Graphpad Prism 7) using a variable slope sigmoidal concentration–response curve, using the Hill equation: E = 1/[1 + (IC_50_/C)h], where E is the inhibition of currents in percentage at concentration C, IC_50_ is the concentration for 50% inhibition of maximum effect and h is the Hill coefficient.

Immunofluorescence

hPCMs were plated into 15 mm-confocal dishes for immunofluorescence staining. After removing culture medium, cells were rinsed once with DPBS, fixed with 4% paraformaldehyde for 10 min, and then permeabilized in 0.1% Triton X-100 for 7 min. The cells were washed 3 times with ice-cold PBS and incubated with a blocking solution containing 1% BSA, 22.5 mg/mL glycine and 0.1% Tween 20 in PBS. After blocking, cells were incubated with primary antibodies in a humidified chamber at 4 °C overnight. Primary antibodies included anti-ACTN2 (Abcam, ab9465, 1:200 dilution) and anti-TNNT2 (Abcam, ab45932, 1:200 dilution) antibodies. After decanting the primary antibody solutions, cells were washed 3 times with PBS, and then incubated with secondary goat anti-rabbit-Alexa Fluor 488 (Thermo, A32731, 1:1000 dilution) or goat anti-mouse-Alexa Fluor 594 (Thermo, A11032, 1:1000 dilution) antibodies for 2-4 h at room temperature in the dark. Finally, cells were washed 3 times with PBS and mounted with DAPI (Thermo, P36981). Samples were imaged on a confocal laser scanning microscope (Leica SP8, Germany) using a 40× water immersion objective, with identical exposure times. Images were further magnified by electronic magnification when necessary. Sarcomere lengths were quantified using the Leica Application Suite X software (LAS_X_Core_3.7.2).

Seahorse analysis

Mitochondrial respiration of hPCMs was characterized as an indicator of cellular metabolism and fitness using a Seahorse XF^e^24 Extracellular Flux Analyzer (Agilent). The Agilent Seahorse XF Cell Mito Stress Test was applied to freshly isolated hPCMs (day 0, D0) and matched PCMs in culture (D5) to measure oxygen consumption rate (OCR) according to the manufacturer’s instructions. In brief, the Seahorse XF Sensor Cartridge was hydrated the day before running the XF Assay by filling each well of the XF Utility Plate with the Seahorse XF Calibrant Solution, was kept in a non-CO_2_ 37°C incubator overnight. On the day of cell isolation, cells were seeded at 1×10^4^ cells/well (for D0) and 2×10^4^ cells/well (for D5) on the day of hPCM isolation. Cell number, as measured by PrestoBlue™ Cell Viability Reagent (Invitrogen™, A13261) on a multi-mode microplate reader (BioTek, Synergy LX) prior to experimentation, was used for OCR data normalization between D0 and D5. Mitochondrial function was analyzed by sequential injections of oligomycin (1 µM), carbonyl-cyanide-4-(trifluoromethoxy) phenyhydrazone (FCCP, 0.25 µM), a mix of rotenone (0.5 µM) and antimycin A (0.5 µM).

Adenovirus infection

hPCMs were seeded into 48-well plates at a density of 3×104 cells/well. Sixteen hours into culture, hPCMs were infected with pADM-CMV-GFP adenovirus (Vigene Biosciences) at a multiplicity of infection (MOI) of 40 (300 μl /well). Medium was replaced 8 h post infection, and cells were imaged at various time points (24, 48, 72 h post infection) using a fluorescent microscope (Leica, DMI4000B). The efficiency of infection was quantified by the dividing rod-shaped cells with green fluorescence by the total number of cells, multiplied by 100%.

Western blotting

Protein was harvested from hPCMs by lysis using RIPA buffer (10 mM Tris-Cl (pH 8.0), 1 mM EDTA, 0.5 mM EGTA, 1% Triton X-100, 0.1% sodium deoxycholate, 0.1% SDS and 140 mM NaCl) supplemented with 1 × protease and phosphatase inhibitor cocktail (Thermo, 78445). Samples were denatured, separated by sodium dodecyl sulphate–polyacrylamide gel electrophoresis (SDS-PAGE), and transferred onto PVDF membranes (Millipore, IPVH00010). The following primary antibodies were used in our study: rabbit anti-phospho-phospholamban (Ser16/Thr17) (Cell Signaling, 8496, dilution ratio1:1000), rabbit anti-phospholamban (D9W8M) (Cell Signaling, 14562, dilution ratio1:1000), mouse anti-GFP antibody [9F9.F9] (abcam, ab1218, dilution ratio 1:1000), and mouse anti-GAPDH antibody (abcam, ab8245, dilution ratio 1:5000). Secondary antibodies included anti-rabbit IgG, HRP-linked antibody (Cell Signaling, 7074S) and anti-mouse IgG, HRP-linked antibody (Cell Signaling, 7076S). Blots were developed using the Clarity™ Western ECL Substrate (Bio-Rad, 1705060) on an imaging instrument (Bio-Rad ChemiDoc XRS+). Densitometric analyses were performed using ImageJ v1.42q.

ATP measurement

For relative measurement of ATP production, 7,000 of freshly isolated cells were pelleted (100 × *g*, 3 min), resuspended in 100 μl cell culture medium, and transferred into white, clear bottom 96-well plates. Then, 100 μl of thawed Cell Titer Glo solution (Promega, G8462) was added to cells, mixed, and incubated for 10 min, protected from light. Luminescence was measured using a multi-mode microplate reader (BioTek, Synergy LX).

To quantitate ATP levels within hPCMs, pre- and post-freeze hPCMs were seeded at a density of 8 ×10^4^ cells per well into white, clear-bottomed 96-well plates. An additional 1 ×10^5^ cells were used for protein quantification, which served as a normalization factor between samples. ATP standard was used to generate a standard curve. Fifty microliters of detergent were added into each sample well, in incubated on an orbital shaker (600-700 rpm) for 5 min to lyse cells and stabilize ATP. Then, 50 l of substrate solution was added and incubated for 5 min with shaking. Plates were dark-adapted for 10 min, and luminescence was analyzed on a multi-mode microplate reader (BioTek, Synergy LX).

Live-cell imaging

For live cell imaging, hPCMs were seeded in 48-well or 96-well cell culture dishes and incubated 37°C, 5% CO_2_. Morphological dynamics of cardiomyocytes were captured using INCUCYTE® S3 (Sartorius AG, Goettingen, Germany) which photographed 4 fields per well at 4-h intervals for a total of 240 hours. Cell length and width measurements were performed using ImageJ (v1.8.0). Cardiomyocyte parameters at the 4 h time point were used as baselines for data normalization.

Drug treatment and determination of IC_50_

CMs were seeded into 384-well plates (Thermo Fisher) at a density of 2.5-3×10^3^ cells per well. For drug treatments, compound stock solutions were diluted in cell culture media to the indicated final drug concentrations. Vehicle (DMSO concentration of 0.5-1% v/v) and untreated (media only) wells were included on each assay plate and used for normalization of plate-specific readouts and quality control. At 48 h post treatment, cell viability was examined using CellTiter-Glo One Solution Assay (Promega, G8462) per manufacturer’s instructions. The bioluminescence was measured using a multi-mode microplate reader (BioTek, Synergy LX). Data represent the mean measurements from triplicate treatments and cell viability was calculated. GraphPad Prism 7 was utilized for curve fitting, IC_50_ calculations, and statistical analysis. Drug information is summarized in the table below:

**Table 2.** Sources of pharmacological agents.

| Drugs | Manufacturer | Catalog no. |
| --- | --- | --- |
| Doxorubucin | Selleck | S1208 |
| Sorafenib | Selleck | S7397 |
| Regorafenib | Selleck | S1178 |
| Ponatinib | Selleck | S1490 |
| Cisapride | Selleck | S5253 |
| Droperidol | Selleck | S4096 |
| Rofecoxib | Selleck | S3043 |
| Rosiglitazone | Selleck | S2556 |
| Tegaserod Maleate | Selleck | S5401 |
| Axitinib | Selleck | S1005 |
| Cabozantinib | Selleck | S1119 |
| Trametinib | Selleck | S2673 |
| Erlotinib | Selleck | S7786 |
| Afatinib | Selleck | S1011 |
| Gefitinib | Selleck | S1025 |
| Imatinib | Selleck | S2475 |
| Lapatinib | Selleck | S2111 |
| Crizotinib | Selleck | S1068 |
| Everolimus | Selleck | S1120 |
| Lestaurtinib | MCE | HY-50867 |
| Mirdametinib | Selleck | S1036 |
| Pazopanib | Selleck | S3012 |
| Cobimetinib | Selleck | S8041 |
| Sunitinib | Selleck | S7781 |


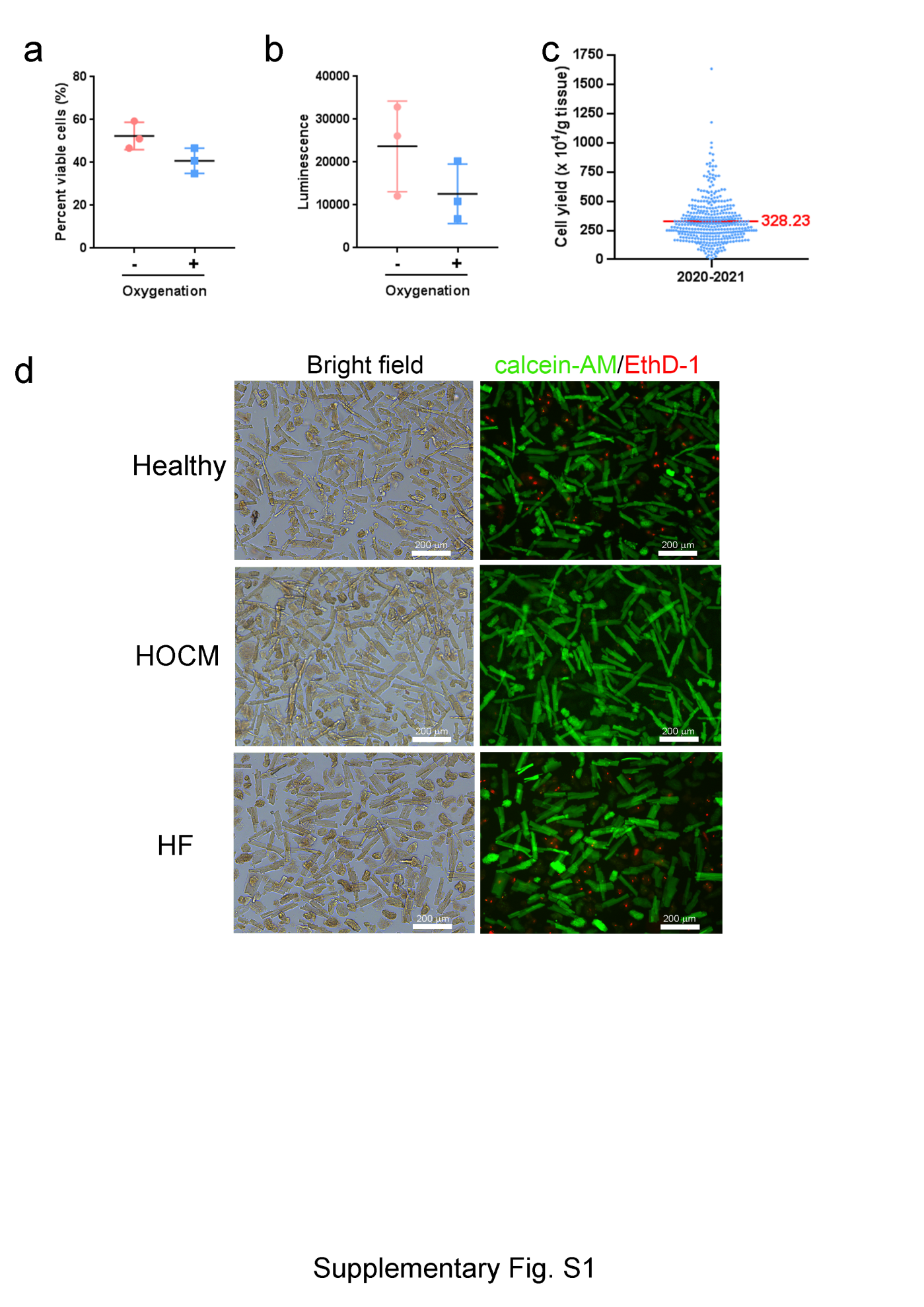
Figure. S1. Isolation quality and yield of hPCMs. a,b Quantification of cell viability by calcein AM/ethidium homodimer-1 staining (a) and assessment of relative cellular ATP levels (b) in freshly isolated hPCMs with or without continuous gassing of 100% oxygen during the isolation procedure. c Average cell yield per gram of myocardial tissue from CABG, MVP, MVR and AVR surgeries during the years 2020 and 2021. d Isolation of ventricular hPCMs from different disease states. Healthy: healthy myocardium; HOCM: hypertrophic obstructive cardiomyopathy; HF: end-stage heart failure. Isolated cells were stained with calcein-AM (live) and EthD-1 (dead) to evaluate cell viability. Scale bar = 200 μm.


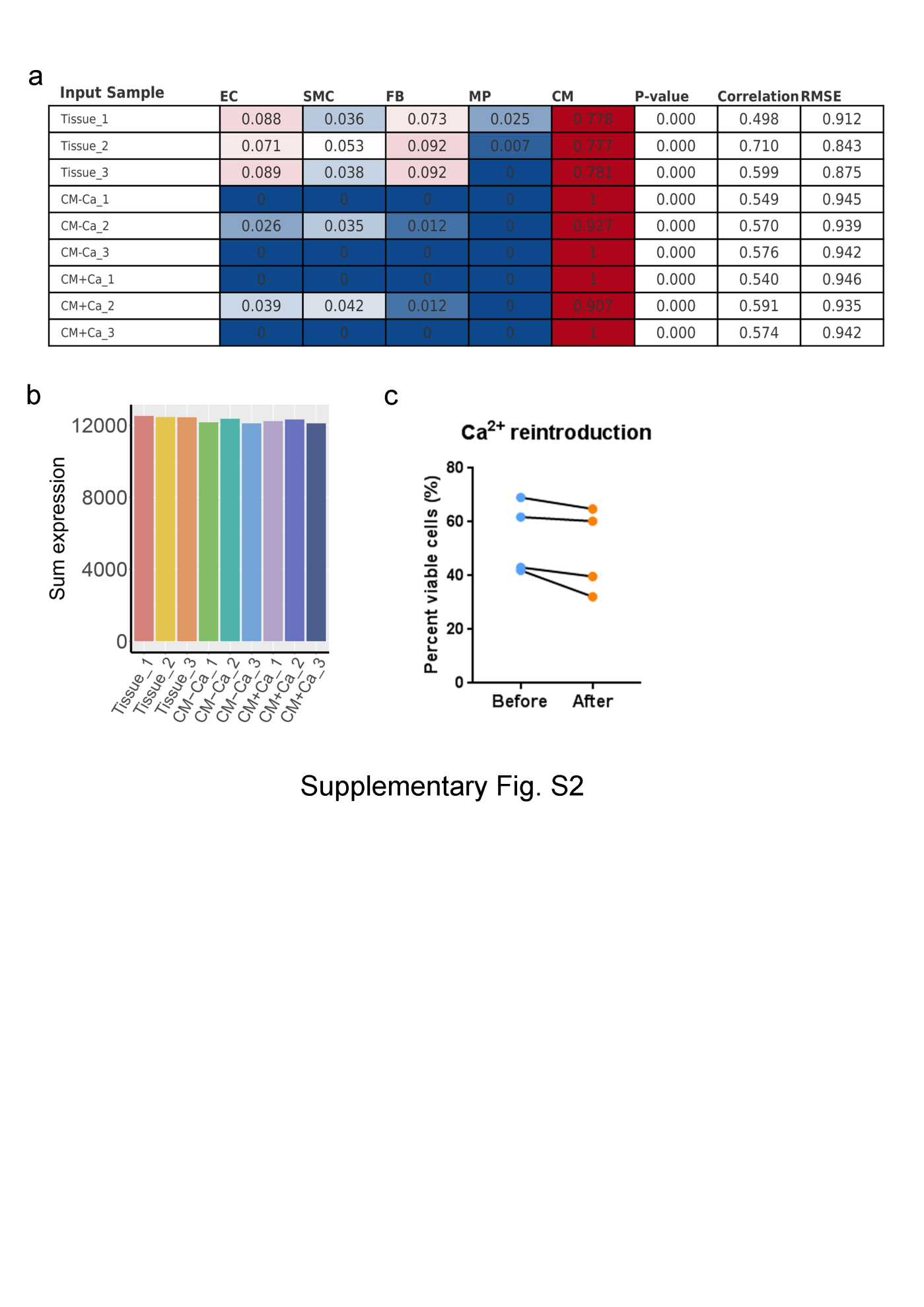


Figure. S2. RNA-seq analysis of freshly isolated hPCMs. a Imputation of cell type-specific gene expression by CIBERSORTx. Tissue, myocardial tissue specimen; CM-Ca, isolated hPCMs prior to calcium reintroduction; CM+Ca, isolated hPCMs after calcium reintroduction. b Calculated CM-specific total gene expression level. c Quantification of cell viability by calcein AM/ethidium homodimer-1staining before and after calcium reintroduction (*P* = 0.1250, Wilcoxon's signed rank test).


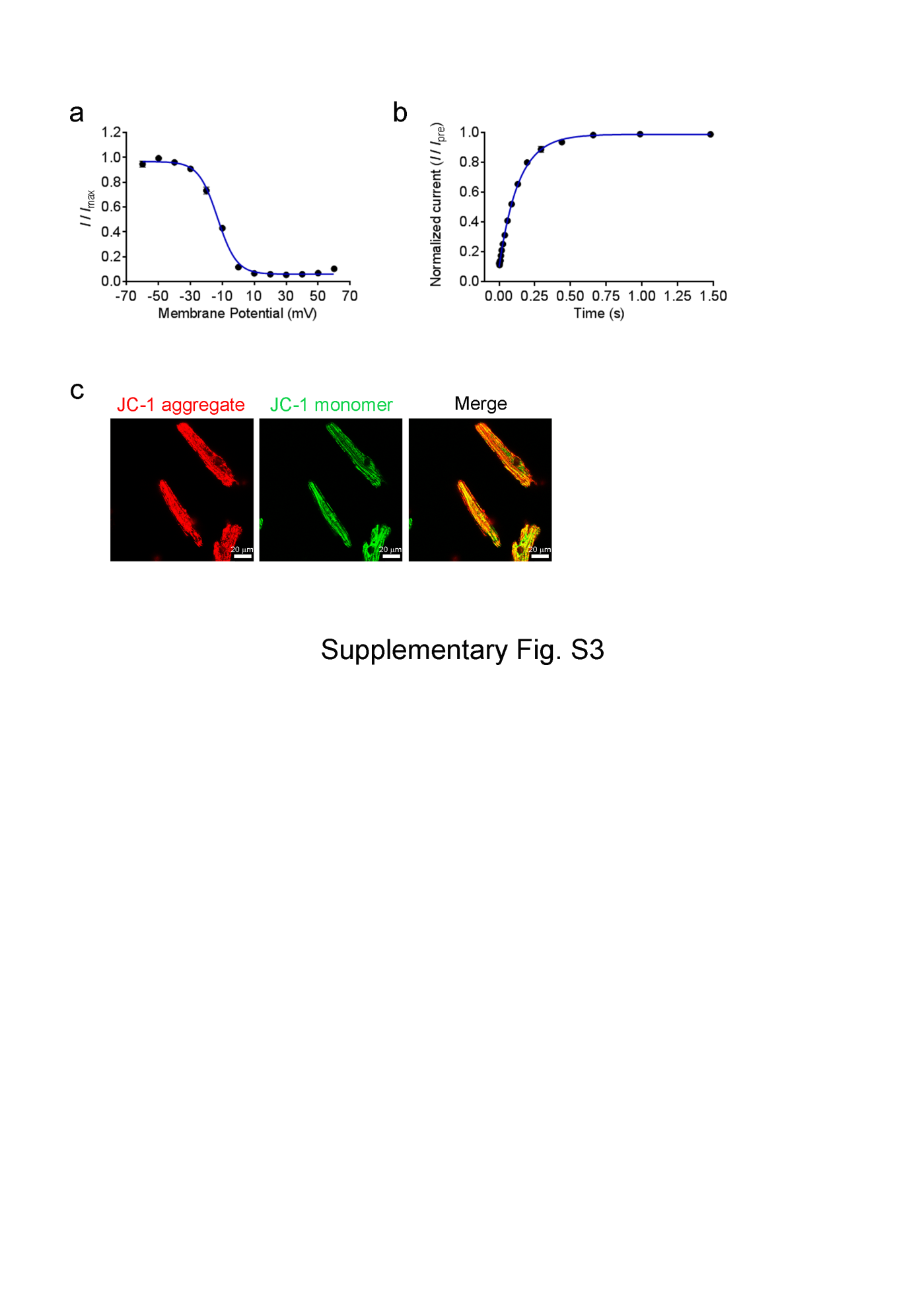
Figure. S3. L-type Ca^2+^-channel parameters and mitochondrial membrane potential of freshly isolated hPCMs. Voltage-dependent inactivation (a) and recovery (b) curves of *I*_Ca,L_ (*n* = 4). c Mitochondrial membrane potential in freshly isolated hPCMs, visualized by JC-1 staining. Results are representative of 3 independent experiments. Scale bar = 20 μm.


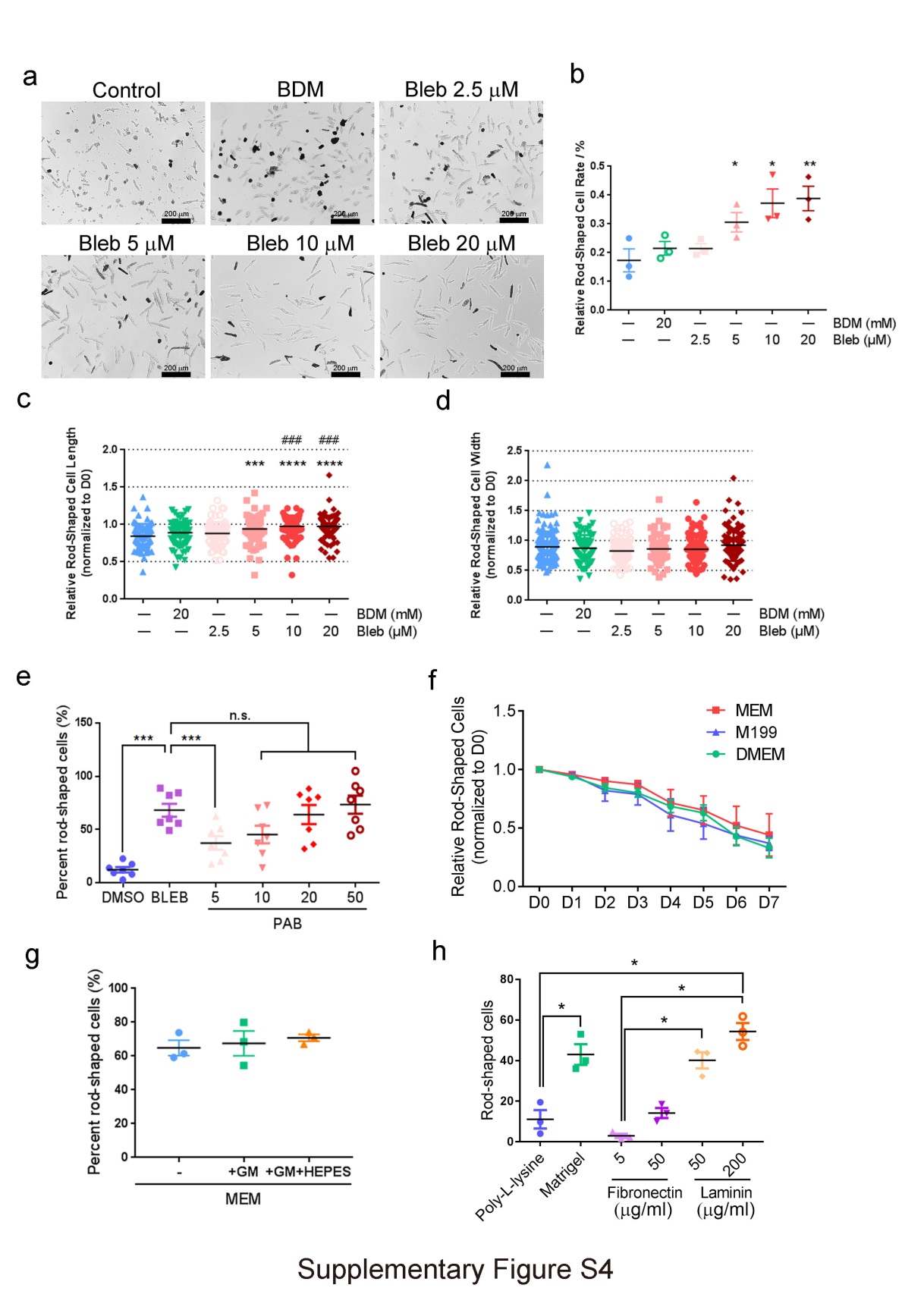


Figure. S4. Optimization of hPCM culture conditions. a Live-cell imaging (D7) of hPCMs cultured with different concentrations of Bleb. Images are representative of 3 independent experiments. b Quantification of the relative percentage of rod-shaped cells in a. c,d Tracking of cell length (c) and cell width (d) changes by D7, normalized to D0. e The effect of para-amino-blebbistatin (PAB) on hPCM culture was evaluated (D7). PAB was used at a series of increasing concentrations: 5, 10, 20 and 50 μM. f Comparison of different types of basal media. g The effect of culture additives GlutaMAX and HEPES on hPCM culture. h Comparison of the pro-survival effects of different surface coating agents. Poly-L-lysine was used at a concentration of 0.01%. Fibronection was used at 5 μg/ml or 50 μg/ml, while laminin was used at 50 μg/ml or 200 μg/ml.


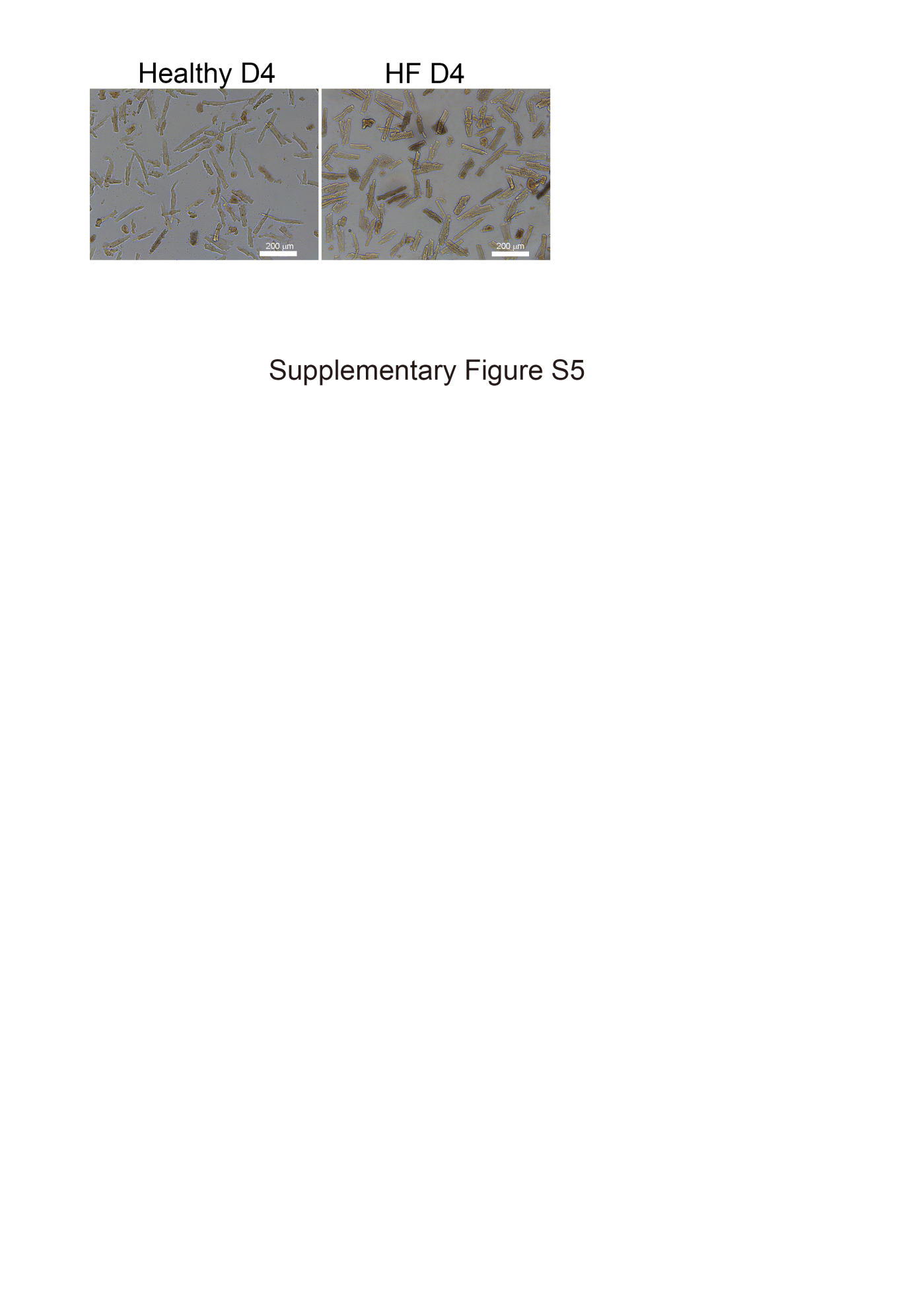


Figure. S5. Culture of ventricular hPCMs from different health states. Bright field images of cultured ventricular hPCMs on day 4 (D4) are shown. Healthy: healthy myocardium; HF: end-stage heart failure. Scale bar = 200 μm


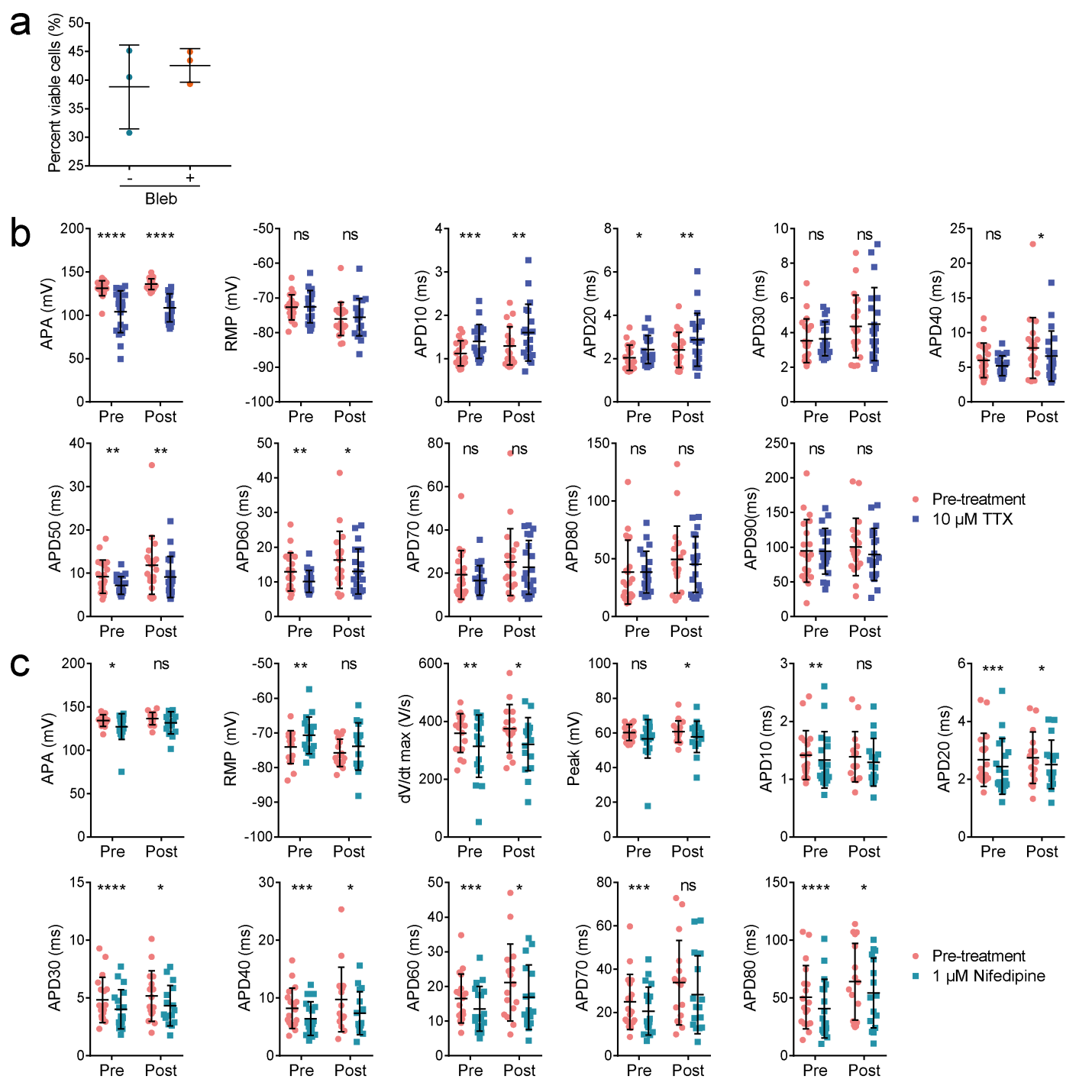
Figure. S6. Evaluation of hPCM quality after cryopreservation. a The effect of Bleb during cryopreservation. a Ten micromolar Bleb was added to the cryopreservation medium, and the effect was measured by cell viability staining immediately upon thawing. b, c Additional action potential parameters of hPCMs before and after cryopreservation. Action potential parameters of hPCMs treated with 10 μM tetradotoxin (TTX) (b) or 1 μM nifedipine (c) both before and after cryopreservation.


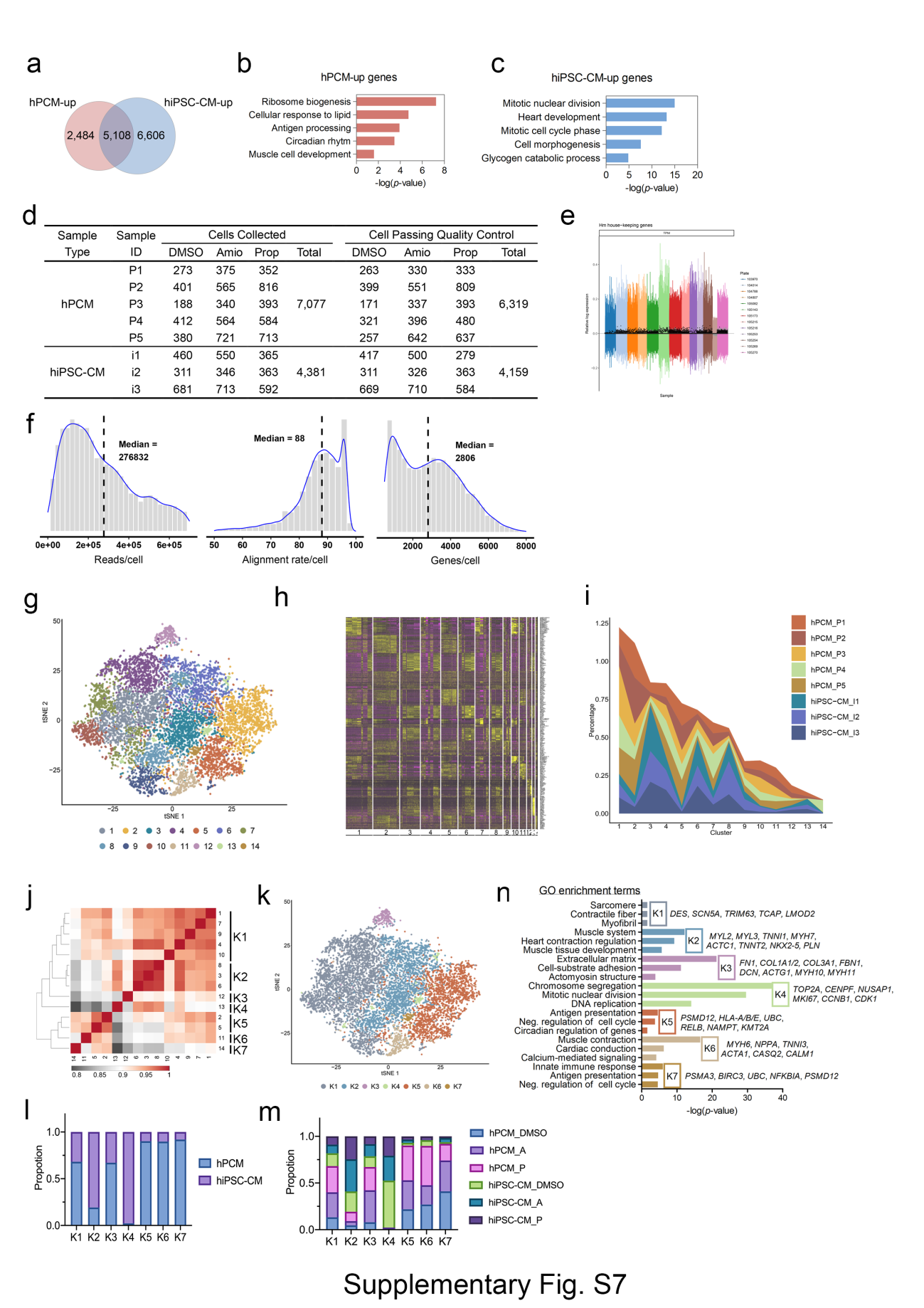


Figure. S7. Population and single-cell drug response of hPCMs and hiPSC-CMs. a Venn diagram showing the numbers of differentially expressed genes (DEGs) between DMSO-treated hPCMs and hiPSC-CMs. b,c Gene ontology (GO) analysis of genes highly expressed in hPCMs (hPCM-up, b) or in hiPSC-CMs (hiPSC-CMs, c), respectively. Selected top categories are shown. d Cell numbers collected from each donor, and after quality control filtering. e Expression of a panel of human housekeeping genes across all chips. f Quality metrics for single-cell RNA-seq data showing distributions of number of reads, alignment rate, and number of genes detected per cell. g *tSNE* visualization of 14 unique cardiomyocyte subclusters identified by *Louvain* clustering. h Heatmap showing 14 cell clusters of differentially expressed genes of all cells sequenced via single-cell RNA-seq. i Contribution of donors to each cluster from h. j *Spearman* correlations showing the transcriptome similarities of the 14 cardiomyocyte clusters in i, which were further grouped into 7 functional K-clusters. k *tSNE* visualization of the 7 K-clusters distribution from b. l, m Bar plots show the cell number proportions in each K-cluster label by cell type (hPCMs and hiPSC-CMs, l) or by cell type and treatment (DMSO, amiodarone or propafenone, m). n GO analysis and representative DEGs (right) in each cell K-cluster. Selected top categories were shown.


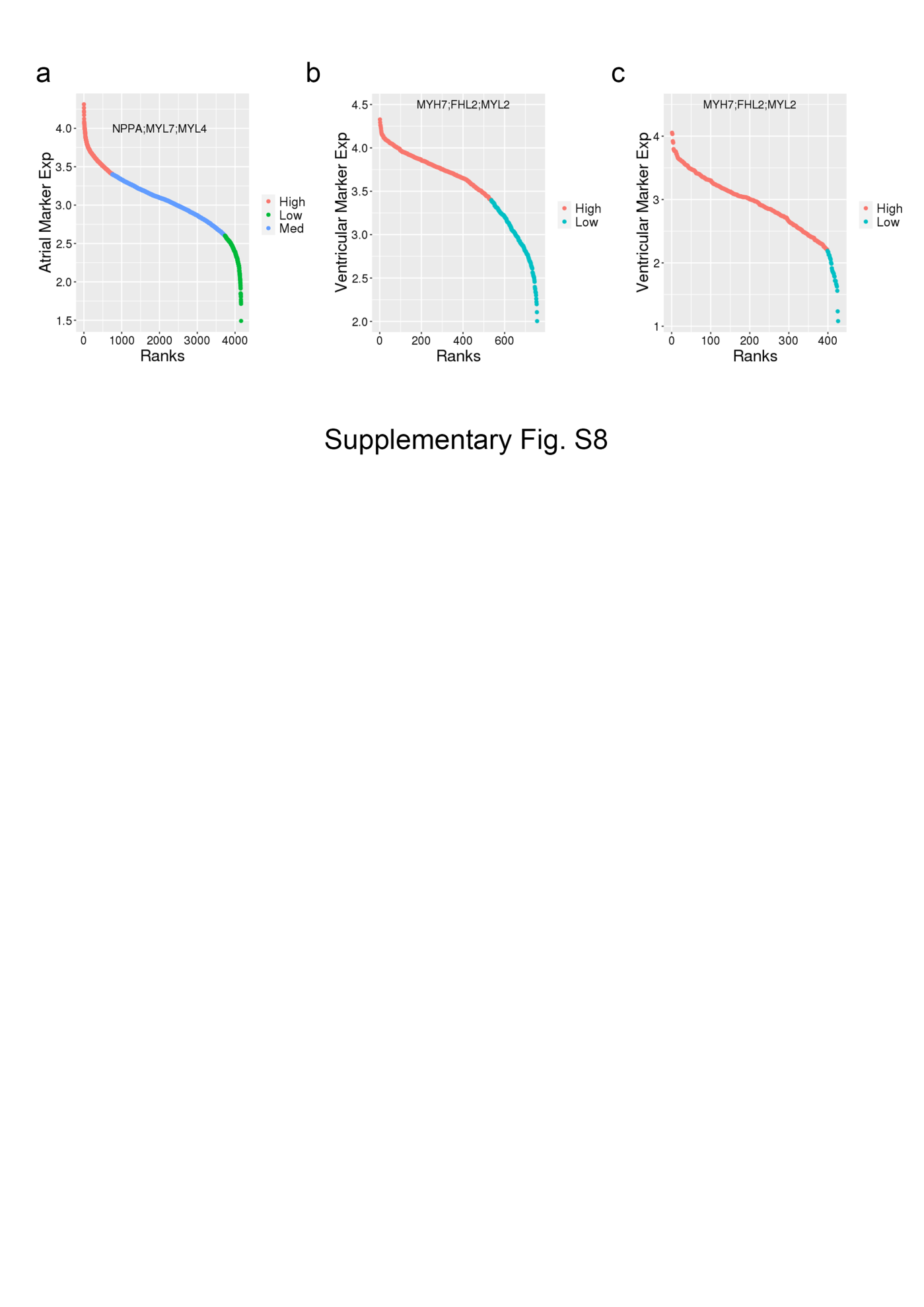
Figure. S8. Selection of atrial and ventricular populations in hiPSC-CMs. a hiPSC-CM cells ranked by the average expression levels of atrial markers *NPPA*, *MYL7* and *MYL4*. The cells with high (red) or low (green) levels of these markers were extracted to b or c, respectively, and then further ranked by the expression of ventricle markers *MYH7*, *FHL2* and *MYL2*, respectively. b, c The cells with low levels of ventricular marker expression in b were identified as ACM (cyan) (b), whereas those with high levels of expression in c were identified as VCM (salmon) (c).

Data S1. (separate file)

DEGs between hPCMs and hiPSC-CMs from buk RNA-seq

GO analysis of DEGs between hPCMs and hiPSC-CMs from scRNA-seq

DEGs among ACMs, VCMs and hPCMs

GO enrichment of DEGs among ACMs, VCMs and hPCMs upon drug treatment
